# Lords of the flies: dipteran migrants are diverse, abundant and ecologically important

**DOI:** 10.1111/brv.70017

**Published:** 2025-04-01

**Authors:** Will L. Hawkes, Myles H.M. Menz, Karl R. Wotton

**Affiliations:** ^1^ Centre for Ecology and Conservation, University of Exeter Cornwall Campus Penryn TR10 9FE UK; ^2^ Swiss Ornithological Institute Sempach 6204 Switzerland; ^3^ College of Science and Engineering, James Cook University Townsville Queensland 4814 Australia; ^4^ Department of Migration Max Planck Institute of Animal Behavior Radolfzell 78315 Germany

**Keywords:** Diptera, migration, Syrphidae, ecological roles, global flyways, seasonal movement, conservation, insect movement, climate change, insect declines

## Abstract

Insect migrants are hugely abundant, with recent studies identifying the megadiverse order Diptera as the major component of many migratory assemblages. Despite this, their migratory behaviour has been widely overlooked in favour of more ‘charismatic’ migrant insects such as butterflies, dragonflies, and moths. Herein we review the available literature on dipteran migration to determine its prevalence, identify key migratory routes and elucidate areas that may prove fruitful for future research. Using 13 lines of evidence to determine migratory behaviour, we determined that species from 60 out of 130 dipteran families show evidence of migration, with Syrphidae fulfilling 12 of these criteria, followed by the Tephritidae with 10. By contrast, 22 families met just two criteria or fewer, underlining the need for more research into the migratory characteristics of these groups. In total, 592 species of Diptera were identified as potentially migratory, making them the most speciose group of insect migrants yet described. Despite this, only 0.5% of dipteran species were found to be migrants, a figure rising to 3% for the Syrphidae, a percentage mirrored by other migratory taxa such as butterflies, noctuid moths, and bats. Research was biased to locations in Europe (49% of publications) and while vast regions remain understudied, our review identified major flyways used by dipteran migrants across all biogeographic realms. Finally, we highlight an unsurpassed level of ecological diversity within dipteran migrants, including ecological roles of huge economic value. Overall, this review highlights how little is known about dipteran migration and how vital their migratory behaviour may be to the health of global ecosystems.

## I. INTRODUCTION

Each year, huge numbers of insects migrate globally to exploit seasonally available resources to increase their reproductive output, and/or escape habitat deterioration, e.g. due to temperature change, disease risk, food quality, or to seek overwintering sites (Chapman, Reynolds & Wilson, 2015; Dingle, 2014; Satterfield *et al*., 2020). Some insects are known to migrate hundreds or even thousands of kilometres in a single journey (Hobson *et al*., 2012), utilising the sun as a compass and favourable winds to facilitate their journeys (Gao *et al*., 2020a; Knoblauch, Thoma & Menz, 2021; Massy *et al*., 2021; Stefanescu *et al*., 2013). Studies of insect migration have focussed mainly on the larger, more ‘charismatic’ insects such as moths, butterflies, and dragonflies (Chapman *et al*., 2015; Menz *et al*., 2022; Stefanescu *et al*., 2013; Wikelski *et al*., 2006; Guerra, Gegear & Reppert, 2014) or agriculturally/economically important species (Jia *et al*., 2022; Jones *et al*., 2019; Li *et al*., 2020). Few have systematically analysed whole migratory assemblages. However, the studies that do exist have revealed a major group of migrants that remain hugely understudied and that are of great ecological importance: the Diptera (Hawkes *et al*., 2022a, 2024).

The Diptera are a massive and megadiverse order of insects, consisting of over 125,000 described species, with some estimates suggesting numbers may easily surpass one million (Wiegmann *et al*., 2011), indicating that a large percentage of all animal species are flies (Marshall, 2012). Dipteran migratory behaviour is poorly known and little studied, despite mass occurrences frequently being observed, including potentially two of the ten Plagues of Egypt described in the book of Exodus: gnats and dog‐flies (Brenton, 1844). Likewise, in Serbian mythology, a legend concerning the death of a she‐demon called a Hala that notes the spring arrival of a plague of Golubatz (*Simulum colombaschense*) flies from the rotting corpse (Караџић, 2005) suggests a basis for this legend within insect migration (Babic, Baranov & Ganslmayer, 1935). Recent systematic studies of insects passing through migratory bottlenecks have shown that Diptera can comprise nearly 90% of the individuals found in migratory assemblages in certain locations (Hawkes *et al*., 2022a, 2024). Ecological assessments of these species suggest that they play a surprisingly large range of ecological roles compared to other migratory insect orders and are of major importance to economies and the natural world (Doyle *et al*., 2020; Hawkes *et al*., 2022a; Wiegmann *et al*., 2011). However, when compared to other insect orders (e.g. Lepidoptera and Odonata), and particularly to vertebrates, very little is known and what information there is, is highly dispersed (Chowdhury *et al*., 2021; Dingle, 2014).

In this review we synthesise knowledge on dipteran migration within the context of the wider migratory assemblage, to understand better the impact of this diverse group on global ecosystems. We collate all known information about dipteran migration globally including which families and species display migratory behaviour. We use this information to identify potential flyways, describe the ecological roles of these migrants, and explore the impacts that anthropogenically induced climate change may have on their migration.

## II. DEFINING MIGRATION

A widely used definition of migration is one based on behavioural characteristics: ‘Migratory behaviour is persistent and straightened‐out movement effected by the animal's own locomotory exertions or by its active embarkation on a vehicle. It depends on some temporary inhibition of station keeping responses but promotes their eventual disinhibition and recurrence’ (Kennedy, 1985, p. 2). For dipteran migrants, and migratory insects in general, there are various viewpoints as to what constitutes migration (e.g. butterfly migration; Chowdhury *et al*., 2021). Herein, we use the broad behavioural definition of migration quoted above, while recognising that currently we can only be certain of migratory behaviour in a few species. Instead of this representing a failure of the definition, this likely reflects a a lack of research into the migratory behaviour of Diptera and other insect taxa (Chowdhury *et al*., 2021).

## III. LITERATURE SEARCH

We searched *Google Scholar*, *Web of Science* and *PubMed* were searched to identify dipteran families with migratory behaviour based on at least one of the following 13 types of evidence [adapted from Chowdhury *et al*. (2021), to fit dipteran migrants better; see online Supporting Information Table S1 for details]: (*i*) seasonal back and forth movement; (*ii*) long‐distance flight; (*iii*) seasonally appropriate directed movement; (*iv*) inability to develop in trapped habitat; (*v*) ability to choose favourable winds; (*vi*) mass arrival; (*vii*) capable of high‐altitude flight; (*viii*) populations with a high rate of gene flow; (*ix*) strong flight capabilities (tethered flight mill); (*x*) orientation within a flight simulator; (*xi*) physiological/morphological changes in the migratory phenotype; (*xii*) seasonal appearance of a disease; and (*xiii*) unable to overwinter (in any state) in trapped location (Table 1).

**Table 1:** Criteria used to establish presence of migratory behaviour in dipteran taxa. Criteria 1–4 form the ‘core four’ most often reported migratory characteristics. These migratory criteria are adapted from those used in Chowdhury *et al*. (2021) and are displayed as a heatmap against their occurrence in different dipteran families in Fig. 1.

Migratory criteria	Description	Example references
(1) Seasonal back and forth movement	Perhaps the strongest indicator of migration, the insects are observed during the springtime and then again in the autumn season. This can be evidenced by peaks in numbers in different migratory seasons (through radar data/citizen science recording, etc.) or by actively seeing the insects moving in one direction during one season, and then back in the opposite direction later in the year.	Florio *et al*. (2020)
(2) Long‐distance flight	Long‐distance flight is important for migratory insects to escape unfavourable habitats.	Hawkes *et al*. (2022a)
(3) Seasonally appropriate directed movement	Directed movement of an insect in a seasonally appropriate direction (e.g. towards higher latitudes in spring, towards lower latitudes in autumn) suggests a preferred flight detection.	Lack & Lack (1951)
(4) Inability to develop in trapped habitat	Larvae are incapable of developing due to unfavourable seasonal climate. This suggests that the adult insects must move away from their current location to lay their eggs in order for their young to survive.	Ashmole *et al*. (1983)
(5) Ability to choose favourable winds	An important factor in insect migration as the winds are used to assist their migrations.	Gao *et al*. (2020a)
(6) Mass arrival	Migratory flies often arrive in large numbers at the same time.	Hawkes *et al*. (2024)
(7) Capable of high‐altitude flight	To migrate, flies often take advantage of higher altitude wind currents. There is little obvious reason for insects to be found consistently at higher altitudes if they are not attempting to move long distances.	Chapman *et al*. (2004)
(8) Populations with a high rate of gene flow	This suggests a high level of movement between populations by individuals.	Mignotte *et al*. (2021)
(9) Strong flight capabilities (tethered flight mill)	To migrate long distances, insects must have strong flight capabilities. This can be evidenced by their performance in a flight mill.	Nilssen & Anderson (1995)
(10) Orientation within a flight simulator	A preferred, seasonally advantageous flight direction in a flight simulator is indicative of migratory behaviour.	Massy *et al*. (2021)
(11) Physiological/morphological changes in the migratory phenotype	This includes any physiological or morphological changes associated with a migratory phenotype. This could include delaying the development of reproductive organs, or changes in morphology between resident and migratory generations.	Doyle *et al*. (2025)
(12) Seasonal appearance of a disease	If the insects are associated with seasonality of a disease, then it is likely that they are acting as vectors – bringing the disease from distant locations.	Nabeshima *et al*. (2009)
(13) Unable to overwinter (in any state) in trapped location	Adult insects are trapped in a region in which they are not capable of surviving the winter (e.g. at high latitudes, above oceans, in high mountain passes, etc.).	Ashmole *et al*. (1983)

To obtain an initial overview, we searched *Google Scholar* for articles published up to February 2023 using the search terms ‘Diptera’ and ‘Migration’ anywhere in an article, and excluded articles with the terms ‘larvae’ and ‘cell’ and ‘development’ to exclude evolutionary development studies. ‘Dispersal’ was also excluded as there are a large number of articles using this term that document small‐scale (≤300 m) movements of Diptera. This methodology yielded 6200 results, of which a minimum of the abstract and results of the first 1000 papers were read carefully for relevance. A provisional list of migratory dipteran families (‘X’) was obtained from these papers, and *Google Scholar* was then searched for specific information on each of these families using the term ‘X migration’. A specific search of dipteran families using ‘X migration’ was also carried out in both *Web of Science* and *PubMed* databases. In *Web of Science*, the search string ‘Diptera migration NOT cell’ yielded 700 results. In *PubMed* the same search string returned 993 results. To identify relevant studies that may not be included in online search databases due to age, manual searches were performed of the reference lists of books on insect migration such as Johnson (1969) and Williams (1958) and of the reference lists of relevant articles. We decided that saturation of the available literature was close to being reached when further searches of *Google Scholar*, *Web of Science*, and *PubMed* using the same search terms repeatedly returned irrelevant articles. In total 344 relevant articles were identified. Most were written in English, but when relevant articles in other languages were found (titles/abstracts automatically translated by the search engines), these were translated and included: French (seven articles), German (six), Japanese (three), and Portuguese, Danish, Chinese and Dutch (one article each).

## IV. PREVALENCE OF MIGRATION

**Fig. 1:**
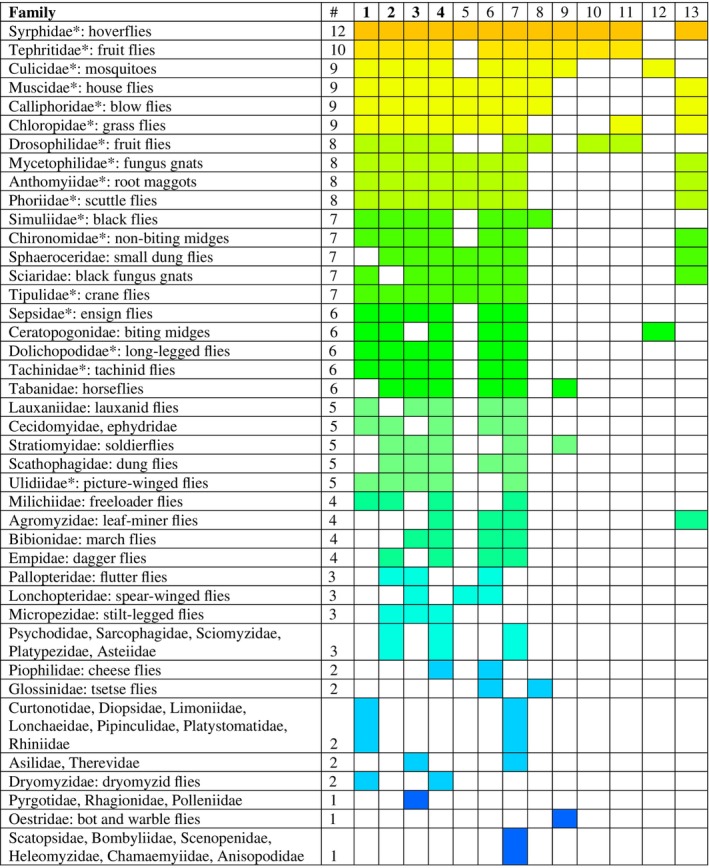
Migratory criteria (see Table 1) fulfilled by the 60 identified migratory families of Diptera. Heat map colours indicate the number of migratory criteria confirmed for each family, with orange being the most (12 criteria fulfilled) and dark blue the least (one criterion fulfilled). # indicates the total number of migratory criteria met. The ‘core‐four’ criteria are: (1) Seasonal back and forth movement; (2) long‐distance flight; (3) seasonally appropriate directed movement; and (4) inability to develop in trapped habitat. Families meeting the core‐four are indicated by an asterisk (*). Other criteria are: (5) ability to choose favourable winds; (6) mass arrival; (7) capable of high‐altitude flight; (8) populations with a high rate of gene flow; (9) strong flight capabilities (tethered flight mill); (10) orientation within a flight simulator; (11) physiological/morphological changes in the migratory phenotype; (12) seasonal appearance of a disease; and (13) unable to overwinter (in any state) in trapped location.

We found evidence for migration behaviour in approximately 47% of all dipteran families (60 out of 130) with data sourced from 344 papers (Fig. 1). Details of the articles and the migration categories for which they provide evidence can be found in Table S2. Syrphidae (hoverflies) were the most studied migratory dipteran family featuring in 48% of articles (recently reviewed by Reynolds *et al*., 2024) and meeting the most migratory criteria: 12/13 (Fig. 1, and Table S2). The Tephritidae (fruit flies) met the second highest number of migratory criteria. Culicidae (mosquitoes) were the second best studied with 17% of the articles and, together with Muscidae (house flies), and Calliphoridae (blow flies and screw worms) and Chloropidae (grass flies), were one of four groups that met nine migratory criteria. All these families, even the minuscule Chloropidae (~2 mm in length), have been recorded as showing the ‘core four’ criteria (Table 1). Studies on Chloropidae have also shown they can choose favourable winds and fly at over 1500 m elevation (Hawkes *et al*., 2024; Glick, 1939).

Drosophilidae (fruit flies), Mycetophilidae (fungus gnats), Anthomyiidae (root maggots) and Phoriidae (scuttle flies) fulfilled eight migratory criteria, including the core four. *Delia platura* (Anthomyiidae) have been recorded migrating in their millions from the Middle East to Cyprus along a northeast trajectory during the springtime, a journey involving an ocean crossing of at least 105 km (Hawkes *et al*., 2022a). Simuliidae (black flies), Chironomidae (non‐biting midges), Sphaeroceridae (small dung flies), Sciaridae (black fungus gnats) and Tipulidae (crane flies) all met seven migratory criteria with Simuliidae, Chironomidae, and Tipulidae meeting the core four criteria. For Tipulidae, there is evidence for seasonal back and forth movement at high altitude above Mali, and records from oil rigs in the North Sea (Hardy & Cheng, 1986; Keaster *et al*., 1996) or in nets on ships in the Gulf of Mexico (Keaster *et al*., 1996) that indicate long‐distance flight across large expanses of ocean. Additionally, Gatter (1977) recorded Tipulidae utilising favourable winds in large numbers to migrate through the mountains of southwest Germany.

Five families fulfilled six migratory criteria with Sepsidae (ant‐like scavenger flies), Dolichopodidae (long‐legged flies) and Tachinidae (tachinid flies), meeting the ‘core four’ criteria. In this group the Ceratopogonidae (biting midges), were recorded undertaking seasonal back and forth movement at high altitude (Florio *et al*., 2020) and long‐distance flight above oceans (Keaster *et al*., 1996).

Six families met five migratory criteria, with only Ulidiidae (picture‐winged flies), fulfilling the core four criteria (Florio *et al*., 2020; Keaster *et al*., 1996; Beebe, 1951).

Four families met four migratory criteria: but none recorded the core four criteria. Despite this, all were recorded in areas unsuitable for larval development. For example, Bibionidae (march flies), were recorded after a migration fallout in snowfields (Ashmole *et al*., 1983). Additionally, Bibionidae have been recorded displaying seasonally adaptive directed movement in large numbers (Beebe, 1951). On May 29th, 1948, Beebe (1951, p. 251) noted a *Bibio* sp. moving through a mountain pass accompanied by a ‘veritable mist of others’. Eight families met three migratory criteria with ‘long‐distance flight’, ‘inability to develop in trapped habitat’, and ‘capable of high‐altitude flight’ the most common criteria met (Sparks *et al*., 1986; Wolf *et al*., 1986; Glick, 1939).

The largest group (12 families) met two migratory criteria, typically ‘high‐altitude flight’. For example, Asilidae (robber flies) have been recorded at medium–high altitude and showed seasonally appropriate directed movement (Glick, 1939; Beebe, 1951). Finally, 10 families met just one migratory criterion, again with ‘high‐altitude flight’ the most common criterion met. Within this group Oestridae (bot and warble flies), met the ‘strong flight capabilities on a tethered flight mill’ criterion with the reindeer warble fly *Hypoderma tarandi* flying for 31.5 h, with a longest continuous flight of 12 h (Nilssen & Anderson, 1995). This capacity must play a role in the insect's life history, likely for following their reindeer hosts on their own extensive migrations.

**Fig. 2:**
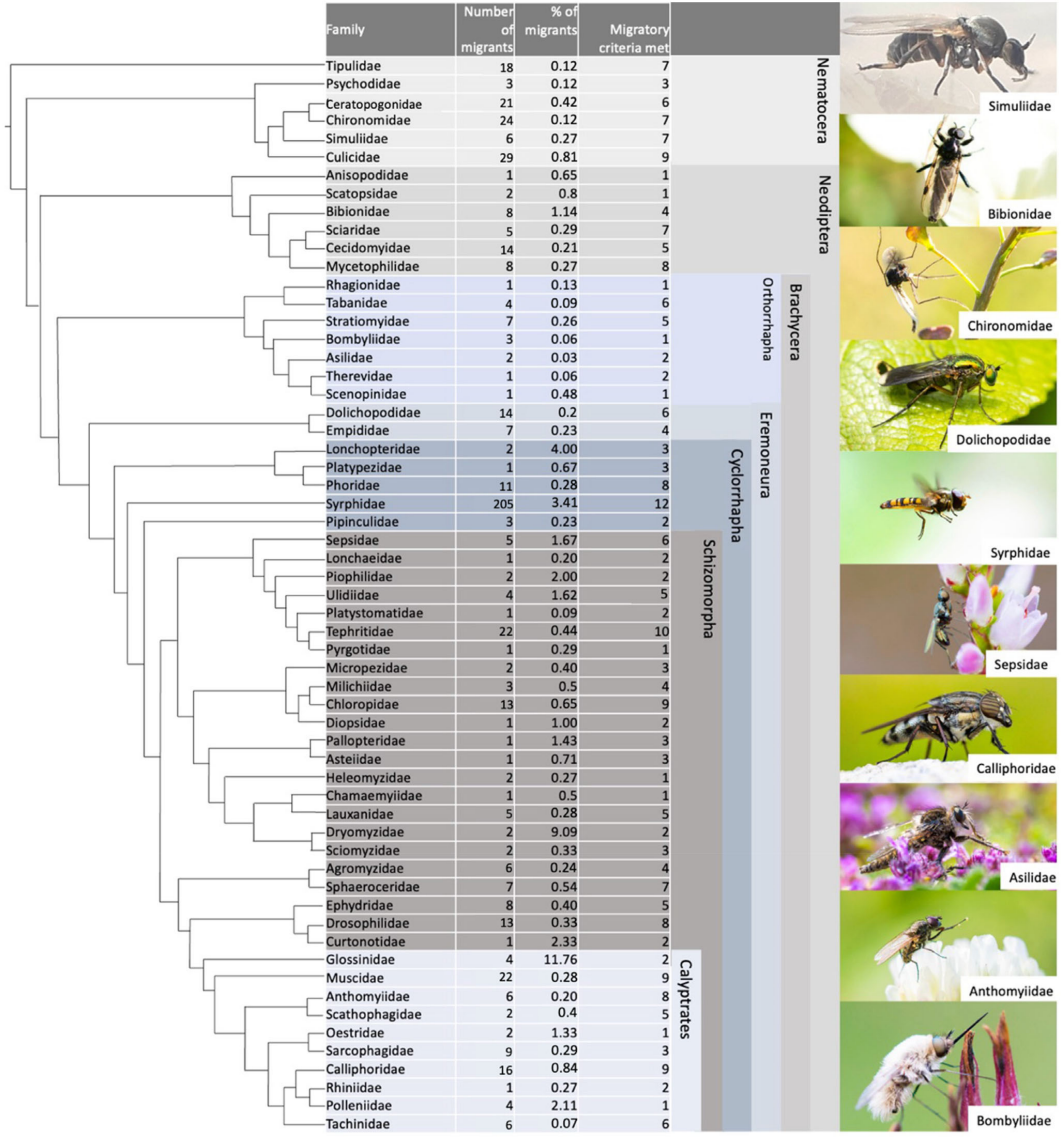
Dipteran phylogeny showing the number of migrant species, the percentage of migratory species in each family, and the number of migratory criteria met. Phylogeny based on Wiegmann *et al*. (2011). All photographs ©Will Hawkes, apart from Simuliidae (©Mehmet Akif Suna).

Evidence for potential migratory behaviour was found for 592 species (see Table S3 for a full species list), representing around 0.5% of identified dipteran species. In the family with the most evidence for migration, the Syrphidae, 205 (3.41%) of the known 6000 species migrate (Fig. 2). Other dipteran families (Glossonidae, Dryomyzidae, and Lonchopteridae) had a higher percentage of migrants, but low species numbers (<150 species) and meet three or less migratory criteria. Interestingly, among butterflies, a well‐studied group of migratory insects, 3% of all species have been diagnosed as migratory (Chowdhury *et al*., 2021) as have 3% of noctuid moths (Alerstam *et al*., 2011) and bats (Fleming *et al*., 2003). While this may imply a consistent pattern across taxa, more research is needed.

## V. MECHANISMS OF FLY MIGRATION

To migrate long distances, flies, like other migrants, rely on a variety of mechanisms to propel and orient themselves on their journeys. Studies performed at migratory bottlenecks suggest that warmer temperatures, dry conditions, and the presence of favourable winds are important factors influencing migration intensity in Diptera (Hawkes *et al*., 2022a, 2024). A radar study across southern Britain showed that in the autumn, Syrphidae actively select winds that aid southward migration (Gao *et al*., 2020a). During the springtime, Syrphidae and other Diptera have been recorded arriving at locations in Europe on winds from the south (Gao *et al*., 2020a; Hawkes *et al*., 2022a,b). Flying higher in favourable tailwinds allows migratory insects to fly faster than their self‐powered airspeed (Chapman *et al*., 2016; Gao *et al*., 2020a). The speed of migratory Syrphidae above southern Britain has been recorded in the springtime at 40.3 km/h, and 35.3 km/h in the autumn (Gao *et al*., 2020a), only a little slower than the speeds of nocturnal migrating moths (spring: 59.7 km/h, autumn: 49.5 km/h) and songbirds (spring: 48.5 km/h, autumn: 43.7 km/h) all speeds faster than their self‐powered flight capability (Chapman *et al*., 2016).

The selection of favourable winds and observations of directed movement points to the presence of a compass system within migratory Diptera. In tethered flight experiments, *Drosophila melanogaster* have been shown to maintain a constant flight heading utilising the sun and polarised light patterns (Warren, Giraldo & Dickinson, 2019; Weir & Dickinson, 2012). However, these headings are arbitrary with respect to a simulated sun and there is no evidence of time compensation as the sun moves across the sky (Warren *et al*., 2019; Weir & Dickinson, 2012). A flight simulator experiment performed on two species of Syrphidae (*Scaeva pyrastri* and *Scaeva selenitica*) caught while migrating through the Pyrenees during the autumn, showed that these species utilise a time‐compensated sun compass as seen in some Lepidoptera and birds, enabling them to maintain their preferred migratory heading even as the sun moves throughout the day (Massy *et al*., 2021; Reppert, 2007; Åkesson & Bianco, 2017). The status of such a compass in other migratory Diptera remains to be investigated.

In addition to using environmental cues, Diptera, like other migrants, undergo changes to their physiology during migration (Chapman *et al*., 2015; Bailleul *et al*., 2012 Jenni & Schaub, 2003; Høgåsen, 1988; Southwood & Avens, 2010; Luschi *et al*., 2007). These changes allow them to store energy and prepare for the long journey ahead. For example, flies will increase their fat stores before migrating, which provides them with the energy they need to fly long distances (Hondelmann & Poehling, 2007). The importance of this has been highlighted in flight mill studies in *Episyrphus balteatus* which demonstrate fuel reserves are a key determinant of distance flown and indicate that resource availability may play a major role in the success of migratory movements (Massy *et al*., 2024). Changes in morphology and size are also common in insect migrants. While little evidence currently exists for dipteran migrants, *E. balteatus* follows a general trend for larger migrants with additional sex‐specific female‐weighted lower wing loading and enhanced flight capacity over male migrants of the same species (Menz *et al*., 2019b; Doyle *et al*., 2025).

A study on the transcriptomes of non‐migratory summer and migratory autumn individuals of *E. balteatus* trapped in a high‐altitude Pyrenean pass revealed over 1500 genes showing strong evidence for differential expression between these generations (Doyle *et al*., 2022). Analyses of these genes revealed a remarkable range of roles in metabolism, muscle structure and function, hormonal regulation, immunity, stress resistance, flight and feeding behaviour, longevity, reproductive diapause, and sensory perception, all of which are key traits associated with migration and migratory behaviour (Doyle *et al*., 2022). Several of these factors, particularly those involved in the circadian system, flight and hormonal control are shared with lepidopteran migrants. This suggests a common genetic basis to these traits and highlights the value of dipteran models for developing a comparative evolutionary framework of migration (Doyle *et al*., 2022).

## VI. GLOBAL DISTRIBUTION AND FLYWAYS

**Fig. 3:**
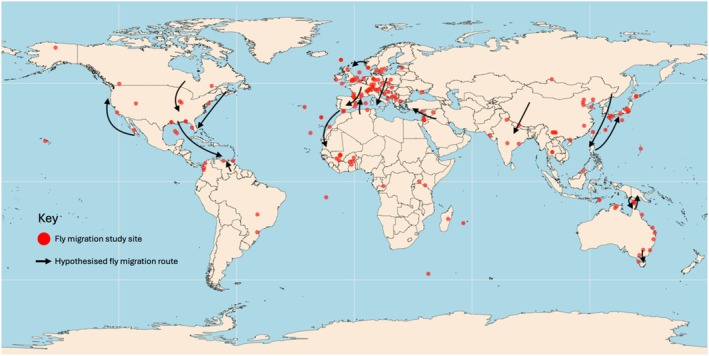
The geographic distribution of dipteran migration studies and migratory flyways. Red dots represent locations of the dipteran migration studies identified in this review. Black arrows represent hypothesised migration routes based on known migrations from the published literature. These arrows represent likely broad fronts of movement and may be further influenced by geography, innate compass senses, and wind patterns.

We found a global distribution of migratory behaviour in Diptera (Fig. 3). Records were recovered from all continents including, surprisingly Antarctica, with the calliphorid *Calliphora croceipalpis* identified as a likely migrant to the sub‐Antarctic Marion Island, 1700 km from South Africa, the closest non‐snow‐covered landmass (Chown & Language, 1994). The data point to a bias of European migration records, which make up 49% of publications, followed by Asia (13%), North America, Africa, and Australasia (all 10%) (Fig. 3). Location‐specific information on the corridors used by these migrants is of key importance for future research, especially given the highly diverse ecological roles of Diptera. Below, we focus on each geographic region where fly migration is known to occur and on routes taken, which we refer to as ‘flyways’ in recognition of the geographic, ecological and climatic features that constrain movement to particular pathways. However, we recognise that much work is required to characterise these routes fully and they remain only hypotheses based on known migrations from the published literature.

### (1) Eastern and western seaboard flyways of North America

On the eastern seaboard of North America, southward migration of Diptera during the autumn was recorded multiple times between 1915 and 1926, with calliphorids (*Cochlyomia macellaria*, *Calliphora vicina*, *Phormia regina*), muscids (*Stomoxys calcitrans*) and syrphids (*Eristalis tenax*) reported moving south in their ‘thousands’ (Shannon, 1926, p. 202). No further records have been published since then, and the status of these movements remains currently unknown (Menz, Brown & Wotton, 2019a). However, recent isotopic studies on the syrphid *Eupeodes americana* suggest that these flies can travel up to 3000 km from Canada to Alabama, indicating that a flyway down the eastern part of North America may still be well‐utilised by migratory Diptera (Clem, Hobson & Harmon‐Threatt, 2023) along with other migratory insects (Howard & Davis, 2009; Reppert & de Roode, 2018; Wikelski *et al*., 2006; Hallworth *et al*., 2018). By contrast, since the observations of Shannon (1926), only one migratory movement has been identified on the western seaboard, a northward movement of presumed *Eupeodes* sp. (Syrphidae) numbering in the hundreds of thousands in just half an hour, recorded on the west coast of California in April 2017 (Menz *et al*., 2019a). It is likely that migratory Diptera regularly move north in the springtime and southwards in the autumn to exploit seasonal resources in North America. Citizen science data for *Eupeodes* (Syrphidae) shows that these flies move from lower latitudes during the winter months to higher latitudes during the springtime, suggesting seasonal long‐distance movements along this flyway (Menz *et al*., 2019a).

### (2) Cross Caribbean flyway

Vast quantities of migratory insects including 17 families of Diptera have been recorded flying south in the autumn through the Portachuelo Pass, Venezuela (Beebe, 1951), along with many migratory bird species (Sainz‐Borgo, Miranda & Lentino, 2020). The Portachuelo Pass runs north–south and opens towards the Caribbean Sea, collecting any insects flying across the ocean. In the late 1940s, insect migration was so plentiful that researchers had to wear glasses to protect their eyes from the swarms (Beebe, 1951). Although no insect‐related studies have occurred at the Portachuelo Pass since then, more recent studies have recorded a variety of migratory Diptera (26 families) alighting on ships and oil rigs in the centre of the Gulf of Mexico, indicating that dipteran migration may occur across the Caribbean Sea (Keaster *et al*., 1996; Sparks *et al*., 1986).

### (3) Western European flyway

Diptera migration has been best studied along the Western European flyway, although even here information remains sparse and restricted to just a few key sites. Long‐term, whole‐assemblage studies have been performed on migratory Diptera using suction traps in the UK, and mountain bottleneck studies in Germany, the French/Swiss Alps, the Pyrenees, and the Czech Republic (Aubert, Aubert & Goeldlin, 1976; Chapman *et al*., 2004; Gatter *et al*., 2020; Hlaváček, Lučan & Hadrava, 2022; Lack & Lack, 1951; Snow & Ross, 1952; Williams *et al*., 1956; Hawkes *et al*., 2024), and there have been many observations of migratory Diptera made from locations in the far north such as Norway and the North Sea, as well as south to the tip of Gibraltar (Ebejer & Bensusan, 2010; Hardy & Cheng, 1986; Jensen, 2001; Nielsen, Andreassen & Leendertse, 2010). A four‐year study at a Pyrenean mountain pass in the autumn revealed 12 families of Diptera migrating south (Hawkes *et al*., 2024). Radar and flight simulator studies have revealed directional movements of migratory Diptera, detailing a SSW bias in their autumnal movements (Chapman *et al*., 2010; Gao *et al*., 2020a; Massy *et al*., 2024). This suggests that migratory Diptera found in Western Europe in the Autumn will be funnelled down into the Iberian Peninsula from large swathes of Europe, before potentially crossing into northern Africa *via* the straits of Gibraltar (Ebejer & Bensusan, 2010).

The majority of migration studies on Diptera have been performed in the autumn, but hints at their springtime routes are available. Large numbers of migratory Syrphidae have been found in the dunes in Gibraltar during the springtime, having just crossed the straits from Africa, a crossing well known for the large number of birds migrating between the continents (Ebejer & Bensusan, 2010). In 2022, large numbers of migratory Diptera, primarily Syrphidae, were found washed up on a beach in SW France, with wind analyses suggesting they were moving north over the Mediterranean before drowning due to a storm (Fisler & Marcacci, 2022). In the same year, large numbers of Syrphidae arrived on the Isles of Scilly, UK, with wind analysis suggesting that they took off over 200 km away in western France (Hawkes *et al*., 2022b). *Culicoides obsoletus* (Ceratopogonidae) fly populations, which spread bluetongue and Schmallenberg viruses, were found to have high levels of gene flow and no genetic structuring at the scale of France during the springtime, suggesting movement during this period (Mignotte *et al*., 2021). Further illumination of the routes may come from ambitious studies such as MoveInEurope, which operates a series of radars across Western Europe, as well as monitoring the routes birds take to understand if they are migrating along with insects to ensure a food source during the journey (Haest *et al*., 2024).

### (4) Eastern European flyway

The best evidence for an Eastern European flyway is from springtime studies reporting millions of Diptera (15 families) moving from the Middle East to Cyprus over at least 105 km of ocean (Hawkes *et al*., 2022a). Many bird species have been found to use this route too, a large number migrating from Eastern Africa before following the Middle Eastern coast (Pedersen, Thorup & Tøttrup, 2019). Linking of the fertile regions of the Middle East by migratory Diptera in the springtime likely has major importance to eastern European countries in terms of nutrient and pollen transfer (Doyle *et al*., 2020; Hawkes *et al*., 2022a; Satterfield *et al*., 2020). During the Autumn, many migratory birds are known to utilise the Georgian corridor to migrate southwards (Verhelst, Jansen & Vansteelant, 2011). The Georgian corridor area, to the best of our knowledge, has not been studied for dipteran movements, but often insect flyways mirror those of birds suggesting it is a location worthy of further study.

### (5) Himalayan flyway

The areas north of India and the Himalayas such as Siberia, Mongolia, western China, and Kazakhstan are extensively fertile, but only seasonally during the summer months (Shpedt *et al*., 2019). Therefore, these represent locations migratory Diptera can use to exploit seasonal resources before returning to the Indian subcontinent during the winter months. Isotopic studies from dragonflies captured in the Maldives suggest that they originated in southern Siberia, signifying huge distances are covered by migratory insects using this flyway (Hobson *et al*., 2012). The great geographic barrier of the Himalayas creates migratory bottlenecks as the migrating animals are directed through mountain passes because of the winds and topography (Gatter, 1980; Westmacott & Williams, 1954). Therefore, identification of these mountain pass bottlenecks could enable easier monitoring of migratory behaviour. A few have been identified but not systematically sampled, providing only indications of a long‐distance movement of migratory Diptera. *Episyrphus balteatus* (Syrphidae) were recorded flying through a Nepalese pass at 3700 m altitude, while various other Syrphidae have been seen migrating through the Thorong La pass at 5416 m altitude (Gatter, 1980; Westmacott & Williams, 1954). While only a handful of studies on migratory Diptera exist from these locations, it is expected to be a highly fertile area for future study.

### (6) African movements

The size and considerable variety of habitats within the African continent makes it likely there are a great deal of dipteran migration routes, although little is known about them. A recent study based on the normalised difference vegetation index (NDVI) showed that the most suitable overwintering habitat for European‐summering painted lady (*Vanessa cardui*) butterflies is within the sub‐Saharan Sahel region (Hu *et al*., 2021) while field data and ecological niche modelling indicates the Afrotropical region (Talavera *et al*., 2023). Migratory Syrphidae have been found crossing the Straits of Gibraltar during the springtime suggesting that insects from Africa do recolonise Europe on their return migration (Ebejer & Bensusan, 2010). NDVI analysis in the Middle East suggested that the numbers of migratory Diptera, like the painted lady butterflies, are also correlated with increased vegetation growth (Hawkes *et al*., 2022a). Therefore, if Diptera follow similar patterns to these butterflies, they too may be crossing the Sahara to reach more favourable Sahel regions. While, to the best of our knowledge, no direct evidence is available for migratory Diptera moving this far, the Bedouin people living at the Bawiti oasis area of Egypt see large numbers of migratory flies moving south in the autumn and north in the spring each year (Mohammed Khozam, personal communication). South of this area, the Sahara Desert continues until the Sahel region of Sudan, the next suitable overwintering habitat for these insects. This suggests that European dipteran migrants may continue across the Sahara on their spring and autumn migrations, making their journeys even more remarkable, but this requires confirmation.

In West Africa in Mali, a total of 28 families of Diptera including Anthomyiidae and Calliphoridae have been recorded making seasonal back and forth movements at altitudes from 40 to 290 m (Florio *et al*., 2020). Some of these species are likely long‐distance migrants that crossed the Sahara, but as the study was primarily nocturnal (aerial traps were open from 17:00 to 07:30 h) it is possible that many diurnal dipteran migrants were missed. Other migration routes in Africa include the annual arrival of Simuliidae flies to the Volta River basin in West Africa from distant source areas with the onset of the migration season (Garms, Walsh & Davies, 1979). Wind patterns also move large quantities of mosquitoes around West Africa, with the West African monsoon winds enabling large numbers of dipteran migrants to exploit the seasonal resources created by the monsoon rains (Dao *et al*., 2014; Huestis *et al*., 2019; Parker *et al*., 2005).

Eastern and southern Africa have even fewer studies than west Africa. However, there is some evidence of dipteran migrants (Glossinidae) arriving with the rains from long distances in Kenya (Brightwell *et al*., 1997). This suggests that flies here too are utilising the regular seasonal patterns of monsoon winds to migrate, and it is likely that far more taxa are also using these meteorological conditions to exploit seasonal resources in the region (Funk *et al*., 2016). Africa is an understudied region in terms of dipteran migration, but there is little doubt there are many migration routes yet to be discovered.

### (7) East Asia to SE Asia

The East Asian Insect Flyway has recently been characterised by Hu *et al*. (2024) with a focus on migratory lepidopteran and planthopper pests. The flyway covers mainland Southeast Asia and the Philippines in the south, through East China and adjacent parts of Mongolia, to the Russian Far East and Japan in the north (Jia *et al*., 2022; Liu *et al*., 2019; Kurahashi, 1997; Nabeshima *et al*., 2009). Dipteran migrants in this flyway have been identified by long‐term studies on Beihuang, a small, isolated island in the Bohai Strait, NE China that included trapping, trajectory analysis, and intrinsic markers, and revealed that *E. balteatus* (Syrphidae) exhibit seasonal back and forth latitudinal movement, passing the island each year on long‐distance migration (Jia *et al*., 2022). Population genetics studies have also revealed that *Eupeodes corollae* (Syrphidae) shows little differentiation in its population across the whole of China, suggesting regular long‐distance movements that maintain gene flow across the whole geographic area (Liu *et al*., 2019). Migration to the Japanese islands from the Asian mainland may also be occurring regularly. Reports have been made of groups of *Calliphora nigribarbis* (Calliphoridae) flies arriving on southern Japan from the Korean peninsula, some 300 km to the NW during the autumn migration season (Kurahashi, 1997). Based on phylogenetic analysis of Japanese Encephalitis Virus (JEV) strains found in Japan, it has been determined that at least some strains originated in Vietnam and China's inland region, while others originated in Shanghai, China (Nabeshima *et al*., 2009). It has been suggested that the mosquito vectors of this disease migrate to the area regularly, brought from SE Asia by a seasonal low‐level jet stream during the rainy season [which also brings the brown leafhopper (*Laodelphax striatellus* ‐ Hemiptera) to Japan] and on westerly winds from mainland China (Nabeshima *et al*., 2009).

### (8) Oceania

Like many areas, Australian migratory Diptera are poorly studied. A flyway of various species seems to exist between SE Asia and Northern Australia, especially between Papua New Guinea and Queensland across the Torres Strait. Mosquitoes are thought to enable the regular ingress of JEV into Australia from Papua New Guinea, utilising favourable winds (Ritchie & Rochester, 2001). Similar movements are known of *Culicoides* sp. (Ceratopogonidae) as vectors of diseases including Blue Tongue between Indonesia, Papua New Guinea and Queensland (Eagles *et al*., 2014). Movements of *Melangyna* sp. (Syrphidae) also have been recorded across the Bass Strait between Tasmania and mainland Australia during the springtime (Hill, 2013), although given the size and climatic variability of the Australian continent, these SE Asia–Australia and Australia–Tasmania flyways are unlikely to be linked. A citizen science study of Syrphidae in Australia showed that there were major latitudinal movements throughout the year in four species (*Melangyna viridiceps*, *Simosyrphus grandicornis*, *Eristalinus punctulatus*, and *Eristalis tenax*), a behaviour suggestive of migration (Finch & Cook, 2020) however, further work is needed in Australia to reveal the true geographical range of movements of migratory Diptera. *Eristalis tenax* is a cosmopolitan species and appears in migration studies from Europe and North America and is found in Australia and New Zealand (Finch & Cook, 2020; Hawkes *et al*., 2022a, 2024; Jia *et al*., 2022; Shannon, 1926). The cosmopolitan distribution of these migrants could allow studies into the behaviour and genomics of the same species across multiple continents.

### (9) Other potential flyways

Vast swathes of the globe are understudied in terms of migratory Diptera and there are undoubtedly more species and flyways to be discovered (Fig. 3). No records of Dipteran migration have been found from sub‐equatorial South America. Given that the vast latitudinal difference covered by this landmass will give rise to many seasonal resources to exploit, conditions seem perfect for the presence of migratory Diptera. Similarly, southern Africa is understudied yet has great potential. One method for determining migratory flyways of Diptera is monitoring the routes of migratory birds or the systematic monitoring of insects at likely visible migration points in the landscape. For example, migratory globe‐skimmer dragonflies (*Pantala flavescens*) migrate between India and Africa on monsoon winds (Anderson, 2009) and so smaller dipteran species may also be traversing the same immense distance to exploit the seasonally available conditions created by the monsoons. Genetic studies have revealed that species of Drosophilidae and Tephritidae found in East Africa have their origins in India, likely having been blown across on the seasonal winds (Jacquard *et al*., 2013; Tsacas, 1984). Additionally, large numbers of *Chrysomya megacephala* (Calliphoridae) were recorded arriving on an island in the Maldives suggesting a similar journey to the globe‐skimmer dragonflies (W.L. Hawkes, personal observations). Interestingly, also on the Maldives, parasitic *Forcipomyia* midges were recorded clinging to the wings of migratory globe‐skimmer dragonflies that had presumably just arrived from India (W.L. Hawkes, personal observations), an example of phoretic migration by these dragonfly‐riding flies. These strands of information on migratory Diptera in these areas provide exciting opportunities for further research.

## VII. ECOLOGICAL ROLES

Compared to migratory insects of other taxa, the variety of ecological roles played by the Diptera is unparalleled. For example, the Odonata are found around freshwater habitats with both predatory larvae and adults while the Lepidoptera mostly have terrestrial plant feeding larvae and are pollinators as adults. By contrast, the Diptera occupy an extraordinary breadth of terrestrial and aquatic habits associated with an impressive range of ecological roles, including as pollinators, decomposers, predators, parasites and pests, while exhibiting a highly diverse range of feeding habits. Diptera are recognised as vital in maintaining ecosystems and as ecosystem engineers and keystone species in many habitats (Adler & Courtney, 2019). We identified a diverse range of dipteran migrants numbering 592 species from 60 families. The ecological roles of these species were determined from a variety of sources including guidebooks and scientific publications (Table S3), revealing that 62% of these species were pollinators, 35% were decomposers, 18% were pests, 16% were disease vectors, 10% pest controllers, and all played a role in the transfer of nutrients. The total sum is over 100% as individual species can play multiple ecological roles. Understanding the ecological roles of migratory Diptera is important when considering the impacts of these movements of flies globally.

### (1) Pollinators

An estimated 62% of identified Dipteran migrants function as pollinators. Rader *et al*. (2020) found that Diptera visited 72% of major food crops. Six families of flies visited more than 12 major food crops, Syrphidae, Calliphoridae, Muscidae, Sarcophagidae, Tachinidae, and Bombyliidae, and all included species that are known migrants. Amongst these families, the Syrphidae and the Calliphoridae were the most common visitors (Rader *et al*., 2020). The Syrphidae alone have been found to pollinate 52% of major food crop plants globally with an estimated worth of around US$300 billion per year (Doyle *et al*., 2020; Rader *et al*., 2020).

Migratory pollinators may be exceptionally important to global ecosystems because, unlike more sedentary pollinator species (like most bees) they transport pollen great distances and can link geographically isolated plant populations (Doyle *et al*., 2020; Lysenkov, 2009; Meyer, Jauker & Steffen‐Dewenter, 2009; Rader *et al*., 2011). Evidence for long‐distance transfer of pollen was found for individual *E. tenax* (Syrphidae) and *C. vicina* (Calliphoridae), caught after flying at least 105 km across the eastern Mediterranean from the Middle East to Cyprus with Bug Orchid (*Anacamptis coriophora*) pollen attached to their faces (Hawkes *et al*., 2022a). Further pollen analysis by DNA barcoding revealed that these same *E. tenax* flies were carrying at least seven other species of pollen upon their bodies (W. L. Hawkes & T. Doyle, unpublished data) while data from migratory *E. balteatus* and *E. corollae* caught in the Alps revealed average pollen loads of 10.5 grains per fly (Wotton *et al*., 2019). Pollen can remain viable for many days (Gibernau *et al*., 2003) and these insects are capable of moving hundreds of kilometres in a matter of hours with wind assistance (Hawkes *et al*., 2022b) suggesting that viable pollen can be transferred great distances. Migratory pollinators can be very numerous; it has been estimated that just two species of Syrphidae could transport 3–8 billion pollen grains into southern Britain from the near continent each year, and 3–19 billion pollen grains out to the continent in the Autumn (Wotton *et al*., 2019). Such movements likely have highly significant consequences for long‐range gene flow mediated by insect migration. For example, the movement of pollen may allow increased gene flow between populations which in turn may increase the resistance of plants to inbreeding depression, increase population survival and maintain the health of isolated populations (Luo, Xia & Lu, 2019; Pérez‐Bañón *et al*., 2003). Migratory pollinators may also allow for adaptions by plant populations to counter a warming climate by spreading alleles favourable for disease resistance or drought (Luo *et al*., 2019; Pérez‐Bañón *et al*., 2003). Small islands without the ability to support populations of sedentary pollinators may especially benefit from migratory dipteran pollinators. For example, in the Columbretes archipelago of Spain the migratory syrphid *E. tenax* is known to be the major pollinator species, alongside the migratory calliphorid *Lucillia sericata* (Pérez‐Bañón, Petanidou & Marcos‐García, 2007).

### (2) Decomposers

Migratory animals rely on arriving in areas with suitable seasonal resources (Dingle, 2014). Many species of migratory Diptera are decomposers (e.g. Calliphoridae and eristaline Syrphidae), taking organic matter from a dead organism or an organism's waste and breaking it down into simple organic substances which can subsequently be taken up by other organisms (Losey & Vaughan, 2006). Studies have revealed that migratory Diptera with a major role in decomposition comprise a significant part of the entire migratory assemblage (16% in Cyprus, 33.6% in Pyrenees) (Hawkes *et al*., 2022a, 2024). From Table S3 we estimate that of all known migrant Diptera, 35% play a role in decomposition.

Many dipteran migrant decomposers feed on decaying plant or fungal matter or animal waste. Migratory Calliphoridae such as *L. sericata* (Diakova *et al*., 2018) and the *C. vicina* group lay their eggs on carrion, upon which their offspring feed (Anderson, 2011). These carrion feeders are known to fly great distances (Hawkes *et al*., 2022a) and some of their populations are considered panmictic, suggesting high levels of migration (Diakova *et al*., 2018), therefore nutrients taken from carrion by their larvae can be redistributed across large areas. The Muscidae provide additional important examples. For example, *Musca domestica* larvae are coprophagous and saprophagous, and prefer to live in areas with high microbial and organic contamination (Čičová *et al*., 2012) where they are efficient decomposers. They therefore aid the biodegradation of organic waste, especially within synanthropic conditions (in association with and benefitting from human activities) (Čičová *et al*., 2012). In ideal conditions, larvae produced by 50 *Musca domestica* flies (25,000 eggs) can decompose up to 444 kg of pig slurry, transforming it into organic compost with excellent agronomic potential (Čičová *et al*., 2012). The migratory eristaline hoverfly *E. tenax* is thought to play similar decomposition roles with great potential for aiding waste recycling (Francuski *et al*., 2014; Ecodiptera, 2009). The impacts of migratory Diptera on decomposition efforts globally have not been quantified, but given their abundance in migratory assemblages, could be large. Strategies involving the planting of wildflowers and provision of other habitats for migratory Diptera near areas where decomposition is needed (livestock slurry pits for example) could allow maximisation of the decompositional roles of migratory Diptera.

### (3) Pests

The intensification of agriculture increasingly means there are vast swathes of land dedicated to monocultures globally (Meyer & Turner, 1992). Migratory species need to be able to find resources, and the species that have evolved to use abundant crops or livestock as a food source have been the most successful (Guo *et al*., 2020). Monocultures of crops have led to local simplification of insect biodiversity, reducing populations of their natural enemies, in turn creating conditions suitable for agricultural pests to flourish (Sánchez‐Bayo & Wyckhuys, 2019). Many migratory Diptera are classed as agricultural pests, from Table S3 we estimate this to be around 18% of all known dipteran migrants. For example, some migratory species of Chloropidae, such as *Oscinella frit*, are pests of various cereals, grasses, and spring‐sown maize (El‐Wakeil & Volkmar, 2011; Southwood & Jepson, 1962). In spring 2019, over 15 million *Delia platura* (Anthomyiidae) were estimated to be migrating long distances (minimum 105 km) from the Middle East to Cyprus; this species is a generalist crop pest of nearly 50 plant species (Guerra *et al*., 2017; Hawkes *et al*., 2022a). This was the first record of this species migrating in such large numbers, suggesting either an increase in its abundance or in the prevalence of its migratory behaviour. Species such as the stable fly *S. calicitrans* (Muscidae) are costly pests of livestock (particularly cattle) (Campbell, Boxler & Adams, 2002; Gerry, 2007), the adult flies feeding on the blood of mammals to provide a protein source before laying their eggs (Bishopp, 1913). These flies seasonally recolonise dairy farms (Beresford & Sutcliffe, 2009) and can fly at least 225 km based on mark–release–recapture experiments (Hogsette & Ruff, 1985). *Cochliomyia homininvorax* (Calliphoridae) is a well‐known migrant and a major pest of livestock. Its larvae cause myiasis, burrowing into the flesh of mammals to feed and develop (Costa‐Júnior *et al*., 2019). Methods for controlling these species often include use of pesticides, however rates of pesticide resistance in migratory organisms have been found to be high (Hemingway *et al*., 1997; Raymond & Pasteur, 1996), underlining the need for a greater understanding of the life histories and movement patterns of migratory species to inform management.

### (4) Disease vectors

One of the most important impacts that migratory Diptera have is as vectors of disease, with 16% of identified dipteran migrants thought to play this role. Of all the migratory dipteran families, the mosquitoes (Culicidae) have been the best studied in this regard. Mosquitoes are known vectors of diseases that kill over half a million people globally every year (Bueno‐Marí *et al*., 2022). *Anopheles coluzzii* mosquitoes which are the primary malaria vector have been shown to engage in windborne migration above Africa, travelling up to 300 km in 9 h (Huestis *et al*., 2019). Of other families, the blackfly *Simulium damosum* (Simuliidae) is capable of moving hundreds of kilometres each year on monsoon winds across west Africa, spreading a nematode (*Onchocerca volvulus*) that causes river blindness (Baker *et al*., 1990), and migratory *Culicoides* sp. (Ceratopgonidae) are known to aid the seasonal recurrence of blue tongue disease in Israel each year (Braverman & Chechik, 1993). Within the Muscidae, the stable fly *S. calcitrans* is thought to be able to transfer food‐associated human pathogens from agricultural to urban areas (Mramba, Broce & Zurek, 2007), as well as directly transmitting wildlife diseases (Mihok & Clausen, 1996). Some migratory Diptera are involved in the transmission of plant diseases. For example, the bean seed fly *D. platura* (Anthomyiidae) has been recently discovered to be a major vector of soft rot bacteria (Pasanen, 2020). Another understudied research area is the role that migratory Syrphidae may play in the transfer of diseases that affect honeybees (*Apis mellifera*) and other bee species, such as deformed wing virus, to previously unaffected populations (Fischer *et al*., 2006). However, although the presence of this disease has been identified within migratory *E. tenax*, there was no evidence of viral replication within *E. tenax* or evidence for whether the disease can be actively passed on to the bees (Fischer *et al*., 2006).

Mosquito‐transmitted diseases are generally a major problem within warmer, more tropical, areas where the Diptera involved in vectoring these diseases (e.g. mosquitoes, tsetse flies, and Simuliidae) can occur in abundance (Huestis *et al*., 2019). With global warming, it is predicted that an additional 4.7 billion people will be affected by these diseases by 2070 compared to 1999 numbers (Colón‐González *et al*., 2021). Rising temperatures are predicted to increase the suitability of temperate locations for the survival of these disease vectors. The range expansion of these diseases could be most problematic in areas where the human population is immunologically naïve, or healthcare systems are unprepared (Colón‐González *et al*., 2021). The migratory behaviour of these dipteran vectors increases the complexity of combatting such diseases as a new influx of pathogens is introduced each year with the migratory insects' arrival (e.g. Lebl *et al*., 2015; Riad *et al*., 2017). This necessitates the development of management plans which consider the long‐distance movement of the vectors. Currently, many insect vectors of disease are understudied in terms of their migratory behaviour, and targeted research on their movement patterns could have significant impacts for human health.

### (5) Pest controllers

Many arthropods are pests that cause damage to agricultural crops, and many migratory Diptera are predators upon these pests at some stage in their life histories (Courtney *et al*., 2009). We found that 10% of all migratory Diptera play the role of pest controllers. These pest controllers include many representatives from the Syrphidae, such as the aphidophagous *E. balteatus* and *E. corollae* (Wotton *et al*., 2019) as well as from the Calliphoridae, such as *Stomorhina lunata* which feeds upon locust larvae (Greathead, 1962). Tachinidae are also known to be useful pest controllers as they lay eggs in a variety of insect larvae including those of the Lepidoptera, Coleoptera, Hemiptera, and Symphyta. For example, the migratory *Tachina fera* (Tachinidae) has been used to control populations of the gypsy moth *Lymantria dispar* in forest environments (Davis, 2013). It is presumed that most migrant species are generalists or at least target highly abundant prey sources. As a result of increased agricultural land coverage, species that are classed as pests have generally become more dominant in recent times (Guo *et al*., 2020). Because of this, the migratory Diptera that prey on these pests are increasingly important, especially given the rise of pesticide resistance and the other ecological benefits that migratory Diptera bring to agricultural landscapes (Doyle *et al*., 2020; Hemingway *et al*., 1997; Raymond & Pasteur, 1996).

Of the migratory Diptera, Syrphidae are best studied regarding pest control (Rojo *et al*., 2003). Aphidophagous Syrphidae are common migrants across all continents except Antarctica, making the total impact in terms of pest control by migratory Syrphidae likely to be huge. Many migratory species such as the abundant *E. balteatus* and *E. corollae* feed on aphids as larvae and therefore are beneficial to agriculture. Indeed, both species are available commercially as biological control agents for use in glasshouses (Moerkens *et al*., 2021; Pineda & Marcos‐García, 2008). The larvae of *E. balteatus* and *E. corollae* are voracious predators and it has been estimated that the progeny of flies migrating to southern England during the springtime consume up to 10 trillion aphids each year (Wotton *et al*., 2019). However, the contribution to biological control by all migratory Syrphidae is likely to be much greater, as the impact of other immigrations or generations produced by other migratory species is yet to be calculated.

### (6) Nutrient transfer

It is thought that insect migration represents the most important animal movement annually in terrestrial ecosystems (Hu *et al*., 2016). As migratory Diptera are multi‐generational migrants, when they reach a suitable area, they lay their eggs and die (Chapman *et al*., 2015), hence, all (100%) of these insects have a function in nutrient transport between geographically distant ecosystems *via* carcass deposition (Hu *et al*., 2016; Satterfield *et al*., 2020). The dry body mass of a migratory fly is typically 10% nitrogen and 1% phosphorus – elements which are limiting to plant growth (Elser *et al*., 2000). Therefore, these insects represent a potential source of nutrient influx for ecosystems. Few studies have documented the influence of nutrient transfer by migratory insects, and fewer still have focussed on the Diptera. Wotton *et al*. (2019) estimated that the 4 billion *E. balteatus* and *E. corollae* Syrphidae migrating above southern England each year comprise 80 tons of biomass and will deposit 2500 kg of nitrogen and 250 kg of phosphorus a considerable distance from their source. The entire migratory assemblage moving annually across southern England has been estimated at 3200 tons, 7.7 times the 415 tons of biomass of migrating songbirds, highlighting the huge importance of migratory insects to nutrient transfer (Hu *et al*., 2016). Migratory Diptera are known to be abundant in migratory assemblages, and extrapolating the values calculated for the Syrphidae to all other migrant Diptera, the movement of nutrients each year is likely to be immense.

However, far more research is needed, particularly for high‐latitude environments, where very few organisms can survive the winter months and where the annual, dependable influx of migratory Diptera may provide vital nutrients for the growth and blooming of vegetation. Animals of higher trophic levels that rely on insects as food, such as birds (Tallamy & Shriver, 2021), may depend on the springtime influx of migratory Diptera each year to provide food for their young or to fuel their own migrations. Finally, a large percentage of migratory Diptera may regularly end up drowning in the sea. Migrating Diptera are often trapped on ships far out in the ocean, for example, *Calliphora nigribarbis* and *Aldrichina grahami* (Calliphoridae) were caught 300–450 km off Japan in the Pacific Ocean (Kurahashi, 1991). While some flies may eventually reach shore, many more likely drown in the ocean due to exhaustion or inclement weather conditions. In 2022, large numbers of Syrphidae were washed up on a beach in southwestern France after being caught in a storm and drowning (Fisler & Marcacci, 2022). These perished flies may provide additional nutrients for marine ecosystems (Hawkes *et al*., 2022a).

## VIII. ANTHROPOGENIC STRESSORS ON MIGRATORY DIPTERA

The natural world is under intense pressure from anthropogenically induced changes with migratory species under a range of threats including climate change, habitat loss, and increased pollution (Cooke *et al*., 2024). In many insect taxa both resident and migratory species have undergone precipitous population declines: one study monitoring flying insect biomass in Germany revealed a 76% decline over 27 years (Hallmann *et al*., 2021). Similarly, in the UK the number of insect impacts on car numberplates reduced by 64% in the 18 years since 2004 (Ball *et al*., 2022). When compared to their sedentary counterparts, however, migratory Diptera that have wide habitat ranges and multiple generations throughout the year are thought to be more resilient to the effects of climate change (Biesmeijer *et al*., 2006). Even so, the few studies documenting declines in migratory dipteran populations are still damning. For example, in the last 50 years the number of aphidophagous Syrphidae migrating autumnally through Randecker Maar in the Schwäbische Alb uplands of southwest Germany has declined by 97% (Gatter *et al*., 2020). Such declines may have drastic impacts. For example, North American insectivorous bird numbers have dropped by an estimated 2.9 billion in the last 50 years, compared to non‐insectivorous birds whose numbers have increased by 26.2 million individuals (Tallamy & Shriver, 2021). A recent European study on Syrphidae has predicted the loss of some sedentary species from lowland areas and gains in alpine locations (Miličić, Vujić & Cardoso, 2018). The majority of agriculture is found in lowland regions and so the loss of these insect pollinators could negatively impact crop yields. Migratory species of Syrphidae have high reproductive rates and mobility and, like other insect migrants (Baker, Venugopal & Lamp, 2015; Bale & Hayward, 2010; Zeng *et al*., 2020), could be capable of adapting to climate change, making them particularly important for counteracting crop damage caused by poleward shifts in pests such as aphids (Bebber, Ramotowski & Gurr, 2013).

While insect population declines are due to a variety of factors, a prominent role is given to climate change and habitat loss (Goulson, 2019). Global temperatures are likely due to rise between 2 and 4.9 °C above pre‐industrial levels by 2100 (Raftery *et al*., 2017). Increasing temperatures therefore could result in higher latitude countries receiving more dipteran migrants. Data on migratory Lepidoptera abundance and temperature spanning 113 years have shown that these migrants have become more abundant in the UK with increasing temperatures (Sparks *et al*., 2007). This is thought to be in part because of increased aridity in southern Europe encouraging northward migration (Sparks *et al*., 2007), something that is likely mirrored by dipteran migrants. Increasing temperatures at higher latitudes is also increasing the suitability for overwintering by migrants. This could lead to the loss or rebalancing of migratory behaviour in many dipteran species which tend to be partial migrants (Menz *et al*., 2019b). As a result, the ecological benefits of the migratory behaviour of Diptera detailed above may also be lost. Interestingly, the presence of partial migration, where part of the population remains in the breeding area instead of migrating, in many species of migratory insect may lead to some resilience to climate shifts (Menz *et al*., 2019b). For example, some individuals of the migratory syrphid *E. balteatus* overwinter in parts of central Europe (Luder, Knop & Menz, 2018; Odermatt, Frommen & Menz, 2017; Raymond *et al*., 2014), and can do so at all life stages: eggs, larvae, pupae and adults (Raymond *et al*., 2014). These overwintering animals provide critical early‐season control of aphids colonising crops before migratory individuals have arrived (Raymond *et al*., 2014). With warming climates, we may see an increase in the proportion of individuals and species overwintering and forgoing migration.

Increasing temperatures due to climate breakdown could also lead to phenological asynchronies between taxa. The timing of dipteran migration may be linked to temperature as seen in some migratory butterflies such as the red admiral (*Vanessa atalanta*) (Sparks, Roy & Dennis, 2005); the phenology of first sighting of Syrphidae in the UK has advanced earlier in the year as the planet warms (Hassall, Owen & Gilbert, 2017). Myriad other organisms may rely upon (or are relied upon by) the arrival of dipteran migrants, including passerine birds, or wildflowers that provide a vital food source for migrating Diptera and may themselves rely on Diptera for pollination services (Hawkes *et al*., 2022a; Losey & Vaughan, 2006). If these organisms use day length rather than temperature to entrain their activities, then asynchrony may result, with potentially disastrous impacts (Mayor *et al*., 2017). A literature review on the ecological impacts of temperature‐mediated trophic asynchrony revealed a dearth of studies (Samplonius *et al*., 2021), with those that do exist biased towards terrestrial higher trophic secondary consumer taxa such as birds, and the northern hemisphere (Samplonius *et al*., 2021). Far more research is needed to inform conservation efforts and to understand the possible consequences of phenological change.

The range of many wind‐borne dipteran migrants is expanding in response to increases in temperature, as seen for some *Aedes* mosquitoes, which are important vectors of diseases such as malaria. Wind patterns transporting mosquitoes to high‐altitude settlements in the Himalayan region used to pose no threat to humans as cold temperatures kill mosquitoes (Dhimal *et al*., 2021). However, with global warming, mosquitoes can now survive in these regions and transmit diseases such as malaria, thus posing a serious threat to these communities (Dhimal *et al*., 2021). Increasing temperatures globally mean that higher latitude countries are now at threat from mosquito‐vectored diseases due to better conditions for mosquito survival (Agyekum *et al*., 2021). The response of disease vectors to changes in climatic conditions has significant consequences for predicting and managing outbreaks of disease.

Increased extreme weather events such as droughts or extended periods of rainfall due to climate breakdown also are likely to have negative impacts on migratory Diptera populations due to changes in habitat suitability. Increased drought may cause vegetation to wither prematurely and the eggs of Diptera to dry out and become unviable. Similarly, increased rainfall may be detrimental to Diptera larvae that develop underground, as they can drown in waterlogged soil. Droughts and the loss of moist habitats can lead to a reduction in the availability of suitable breeding sites for many saprophagous species that have semi‐aquatic larvae, such as the syrphid *E. tenax*.

Finally, increased CO_2_ levels have been shown to reduce the amount of nitrogen in plant leaves by 10–30%. To compensate for reduced nitrogen availability, herbivory levels by crop pests (including many migratory Diptera) may increase by 20–90% (Kinney *et al*., 1997; Roth & Lindroth, 1994, 1995), potentially leading to increased crop damage. Very little is known about the response of migratory Diptera to climate change. Research into the ecological roles, range shifts, and declines of these hugely important species is needed so that their impacts can be understood, encouraged or mitigated, particularly in the context of ecosystem and human health.

## IX. CONCLUSIONS

(1) Our analyses of the literature on migrant Diptera reveal a highly diverse set of 592 suspected migrant species from 60 families, many of which appear to be highly abundant, and to migrate in huge numbers.
(2) Dipteran migrants, compared to other migratory insect orders, play an unsurpassed range of ecological roles, marking them out as a major contributing force to the functioning of terrestrial and aquatic ecosystems and the economy.
(3) Compared to other groups, very little is known about migratory Diptera and for many of the migratory families only a single study related to migration was found. We recommend a greater focus on the taxonomic diversity of dipteran migrants in future studies. This will help to understand the ecological roles of these insects as they connect distant landscapes.
(4) We recommend further study using techniques such as monitoring and trapping in migration bottlenecks, stable isotope and pollen analysis, trajectory analysis, flight simulators and flight mills, and NDVI measurements, along with emerging approaches such as radar networks, that can be used to infer behaviour, assemblages, origins, destinations, headings and numbers of mass movements of dipteran migrants.
(5) Many anthropogenically beneficial dipteran migrants are under threat from climate change and other anthropogenic impacts. It is possible that many migratory flies and their behaviour could disappear without being documented unless action is taken.
(6) To conserve these vitally important taxa, it will not be enough to protect or restore habitat at single locations; the entire migratory route must be capable of sustaining these insects, as for other migratory species (Runge *et al*., 2014). Refocusing agricultural, rewilding and conservation practices to ensure landscape connectivity could have the greatest impacts. Understanding the migratory cycles and pathways of these ecologically important species will be key to future conservation measures.

## Supporting information


**Table S1.** Comparison of migratory criteria for Diptera used herein with those used by Chowdhury *et al*. (2021).
**Table S2.** Evidence for fulfilling migratory criteria in each of the dipteran families.
**Table S3.** Evidence for migratory behaviour and their ecological roles in each of the 592 known dipteran migrant species.

## References

[brv70017-bib-0001] * Abajue, M. C. , Ewuim, S. C. & Akunne, C. E. (2013). Insects associated with decomposing pig carrions in Okija, Anambra State, Nigeria. The Bioscientist Journal 1(1), 54–59.

[brv70017-bib-0002] * Adam, Y. , Bouyer, J. , Dayo, G. K. , Mahama, C. I. , Vreysen, M. J. , Cecchi, G. , Abd‐Alla, A. M. M. , Solano, P. , Ravel, S. & De Meeus, T. (2014). Genetic comparison of Glossina tachinoides populations in three river basins of the Upper West Region of Ghana and implications for tsetse control. Infection, Genetics and Evolution 28, 588–595. 10.1016/j.meegid.2014.03.023 24709401

[brv70017-bib-0003] * Adams, R. H. (1941). Stratification, diurnal and seasonal migration of the animals in a deciduous forest. Ecological Monographs 11(2), 189–227.

[brv70017-bib-0004] * Adesiyun, A. A. & Southwood, T. R. E. (1979). Differential migration of the sexes in *Oscinella frit* (Diptera: Chloropidae). Entomologia Experimentalis et Applicata 25(1), 59–63.

[brv70017-bib-0005] Adler, P. H. & Courtney, G. W. (2019). Ecological and societal services of aquatic Diptera. Insects 10(3), 70. 30875770 10.3390/insects10030070 PMC6468872

[brv70017-bib-0006] * Adler, P. H. & McCreadie, J. W. (2019). Black flies (Simuliidae). In Medical and Veterinary Entomology, pp. 237–259. Academic Press, London.

[brv70017-bib-0007] * Agarwala, B. K. (2018). Taxonomic status and additional description of White's Stalked‐eyed Fly Cyrtodiopsis whitei (Curran, 1936) (Diptera: Diopsidae) from India with a key to the allied species and note on its habitat. Journal of Threatened Taxa 10(8), 12035–12043.

[brv70017-bib-0008] Agyekum, T. P. , Botwe, P. K. , Arko‐Mensah, J. , Issah, I. , Acquah, A. A. , Hogarh, J. N. , Dwomoh, D. , Robins, T. G. & Fobil, J. N. (2021). A systematic review of the effects of temperature on *Anopheles* mosquito development and survival: implications for malaria control in a future warmer climate. International Journal of Environmental Research and Public Health 18(14), 7255. 10.3390/ijerph18147255. 34299706 PMC8306597

[brv70017-bib-0009] Åkesson, S. & Bianco, G. (2017). Route simulations, compass mechanisms and long‐distance migration flights in birds. Journal of Comparative Physiology. A, Neuroethology, Sensory, Neural, and Behavioral Physiology 203(6), 475–490. 10.1007/s00359-017-1171-y. 28500441 PMC5522512

[brv70017-bib-0010] * Aksoy, S. , Caccone, A. , Galvani, A. P. & Okedi, L. M. (2013). *Glossina fuscipes* populations provide insights for human African trypanosomiasis transmission in Uganda. Trends in Parasitology 29(8), 394–406. 23845311 10.1016/j.pt.2013.06.005 PMC3772539

[brv70017-bib-0011] * Al‐Dobai, S. , Reitz, S. & Sivinski, J. (2012). Tachinidae (Diptera) associated with flowering plants: estimating floral attractiveness. Biological Control 61(3), 230–239.

[brv70017-bib-0012] Alerstam, T. , Chapman, J. W. , Bäckman, J. , Smith, A. D. , Karlsson, H. , Nilsson, C. , Reynolds, D. R. , Klaassen, R. H. & Hill, J. K. (2011). Convergent patterns of long‐distance nocturnal migration in noctuid moths and passerine birds. Proceedings of the Royal Society B: Biological Sciences 278(1721), 3074–3080. 10.1098/rspb.2011.0058. PMC3158935 21389024

[brv70017-bib-0013] * Aly, M. F. , Abdelrhim, A. S. & Ali, A. M. (2023). Study the application of insecticide program and its impact on the relationship between leaf Miner Liriomyza huidobrensis populations and the early blight disease in potato fields. Journal of Plant Protection and Pathology 14(6), 171–180.

[brv70017-bib-0014] Anderson, G. S. (2011). Comparison of decomposition rates and faunal colonization of carrion in indoor and outdoor environments. Journal of Forensic Sciences 56(1), 136–142. 20840295 10.1111/j.1556-4029.2010.01539.x

[brv70017-bib-0015] * Anderson, G. S. , Barton, P. S. , Archer, M. & Wallace, J. R. (2019). Invertebrate scavenging communities. In Carrion Ecology and Management, pp. 45–69. Springer International Publishing, Cham.

[brv70017-bib-0016] Anderson, R. C. (2009). Do dragonflies migrate across the western Indian Ocean? Journal of Tropical Ecology 25(4), 347–358.

[brv70017-bib-0017] * Arai, S. , Kuwata, R. , Higa, Y. , Maekawa, Y. , Tsuda, Y. , Roychoudhury, S. , Bertuso, A. G. , Phong, T. V. , Yen, N. T. & Etoh, T. (2022). Two hidden taxa in the Japanese encephalitis vector mosquito, *Culex tritaeniorhynchus*, and the potential for long‐distance migration from overseas to Japan. PLoS Neglected Tropical Diseases 16(6), e0010543. 10.1371/journal.pntd.0010543. 35771889 PMC9278767

[brv70017-bib-0018] * Arcella, T. , Hood, G. R. , Powell, T. H. , Sim, S. B. , Yee, W. L. , Schwarz, D. , Egan, S. P. , Goughnour, R. B. , Smith, J. J. & Feder, J. L. (2015). Hybridization and the spread of the apple maggot fly, *Rhagoletis pomonella* (Diptera: Tephritidae), in the northwestern United States. Evolutionary Applications 8(8), 834–846. 10.1111/eva.12298. 26366200 PMC4561572

[brv70017-bib-0019] * Ashmole, N. P. & Ashmole, M. J. (1988). Insect dispersal on Tenerife, Canary Islands: high altitude fallout and seaward drift. Arctic and Alpine Research 20(1), 1–12.

[brv70017-bib-0020] * Ashmole, N. P. & Ashmole, M. J. (1997). The land fauna of Ascension Island: new data from caves and lava flows. Journal of Biogeography 24(5), 549–589.

[brv70017-bib-0021] Ashmole, N. P. , Nelson, J. M. , Shaw, M. R. & Garside, A. (1983). Insects and spiders on snowfields in the Cairngorms, Scotland. Journal of Natural History 17(4), 599–613. 10.1080/00222938300770491.

[brv70017-bib-0022] * Aubert, J. (1962). Observations sur les migrations d'insectes au col de Bretolet (Alpes valaisannes, 1923 m). Mitteilungen der schweizerischen entomologischen Gesellschaft 36, 304–312.

[brv70017-bib-0023] * Aubert, J. (1964). L'activité entomologique de l'observatoire du col de Bretolet. Bulletin de la Murithienne 81, 105–131.

[brv70017-bib-0024] Aubert, J. , Aubert, J.‐J. & Goeldlin, P. (1976). Twelve years of systematic collecting of syrphids (Diptera) at the Bretolet pass (Alps of Valais). Mitteilungen Der Schweizerischen Entomologischen Gesellschaft 49(1/2), 115–142.

[brv70017-bib-0025] * Aubert, J. & Goeldlin, P. (1981). Observations sur les migrations de Syrphides (Dipteres) dans les Alpes de Suisse Occidentale. Mitteilungen Der Schweizerischen Entomologischen Gesellschaft 54(4), 377–388.

[brv70017-bib-0026] * Aubert, J. & Jaccard, M. (1981). La migrations de Syrphides (Dipteres) dans les Jura Vaudois. Mitteilungen Der Schweizerischen Entomologischen Gesellschaft 54(4), 367–370.

[brv70017-bib-0027] * Ayaz, B. & Altunsoy, F. (2024). Effects of heavy metal pollution on population dynamics of another important pollinator insect group: Horseflies (Diptera: Tabanidae). Biological Diversity and Conservation 17(2), 148–155. 10.46309/biodicon.2024.1353718.

[brv70017-bib-0028] Babic, I. , Baranov, N. & Ganslmayer, R. (1935). The Golubatz Fly in 1934. Arch. Tierheilk. 69(3), 205–212.

[brv70017-bib-0029] * Babytskiy, A. I. , Moroz, M. S. , Kalashnyk, S. O. , Bezsmertna, O. O. , Voitsekhivska, O. V. & Dudiak, I. D. (2019). New findings of pest sciarid species (Diptera, Sciaridae) in Ukraine, with the first record of Bradysia difformis. Biosystems Diversity 27(2), 131–141. 10.15421/011918.

[brv70017-bib-0030] Bailleul, F. , Lesage, V. , Power, M. , Doidge, D. W. & Hammill, M. O. (2012). Migration phenology of beluga whales in a changing Arctic. Climate Research 53(3), 169–178.

[brv70017-bib-0031] * Bajerlein, D. , Jarmusz, M. , Gregor, A. & Grzywacz, A. (2022). Diptera (Dryomyzidae, Fanniidae, Muscidae, Piophilidae) associated with pig carcasses in a forest habitat of Poland: sex‐related patterns of visitation and effectiveness of sampling methods. Journal of Medical Entomology 59(2), 514–524. 34984468 10.1093/jme/tjab218

[brv70017-bib-0032] Baker, M. B. , Venugopal, P. D. & Lamp, W. O. (2015). Climate change and phenology: *Empoasca fabae* (Hemiptera: Cicadellidae) migration and severity of impact. PLoS One 10(5), e0124915. 25970705 10.1371/journal.pone.0124915 PMC4430490

[brv70017-bib-0033] * Baker, P. S. , Chan, A. S. T. & Zavala, M. J. (1986). Dispersal and orientation of sterile *Ceratitis capitata* and *Anastrepha ludens* (Tephritidae) in Chiapas, Mexico. Journal of Applied Ecology, 23(1), 27–38.

[brv70017-bib-0034] * Baker, R. H. A. , Baldry, D. A. T. , Boakye, D. & Wilson, M. (1987). Measures aimed at controlling the invasion of *Simulium damnosum* Theobald sl (Diptera: Simuliidae) into the *Onchocerciasis* control Programme area. III. Searches in the Upper Niger Basin of Guinea for additional sources of flies invading south‐eastern Mali. International Journal of Pest Management 33(4), 336–346.

[brv70017-bib-0035] Baker, R. H. A. , Guillet, P. , Seketeli, A. , Poudiougo, P. , Boakye, D. , Wilson, M. D. & Bissan, Y. (1990). Progress in controlling the reinvasion of windborne vectors into the western area of the onchocerciasis control Programme in West Africa. Philosophical Transactions of the Royal Society of London. B, Biological Sciences 328(1251), 731–750. 1976266 10.1098/rstb.1990.0141

[brv70017-bib-0036] Bale, J. S. & Hayward, S. A. L. (2010). Insect overwintering in a changing climate. Journal of Experimental Biology 213(6), 980–994. 20190123 10.1242/jeb.037911

[brv70017-bib-0037] Ball, L. , Still, R. , Riggs, A. , Skilbeck, A. , Shardlow, M. , Whitehouse, A. & Tinsley‐Marshall, P. (2022). The Bugs Matter Citizen Science Survey: Counting insect ‘splats’ on vehicle number plates. policycommons.net.

[brv70017-bib-0038] * Ball, S. & Morris, R. (2015). Britain's Hoverflies: A Field Guide: A Field Guide. Princeton University Press, Oxford, UK.

[brv70017-bib-0039] * Banks, C. J. (1959). Experiments with suction traps to assess the abundance of Syrphidae (Diptera), with special reference to aphidophagous species. Entomologia Experimentalis et Applicata 2(2), 110–124. 10.1111/j.1570-7458.1959.tb02102.x.

[brv70017-bib-0040] * Bänziger, H. & Pape, T. (2004). Flowers, faeces and cadavers: natural feeding and laying habits of flesh flies in Thailand (Diptera: Sarcophagidae, Sarcophaga spp.). Journal of Natural History 38(13), 1677–1694. 10.1080/0022293031000156303.

[brv70017-bib-0041] * Barnes, J. K. (2008). The genus *Atomosia* Macquart (Diptera: Asilidae) in North America north of Mexico. Proceedings of the Entomological Society of Washington 110(3), 701–732. 10.4289/07-073.1.

[brv70017-bib-0042] * Barros, L. M. , Ferreira‐Keppler, R. L. , Martins, R. T. & Gutjahr, A. L. N. (2019). Bionomy of *Hermetia illucens* (Diptera: Stratiomyidae) on decomposing swine carcass in an urban area of Central Amazon. Journal of Medical Entomology 56(3), 681–689. 30759224 10.1093/jme/tjz005

[brv70017-bib-0043] * Bartsch, S. , Bauer, B. , Wiemann, A. , Clausen, P. H. & Steuber, S. (2009). Feeding patterns of biting midges of the *Culicoides obsoletus* and *Culicoides pulicaris* groups on selected farms in Brandenburg, Germany. Parasitology Research 105, 373–380. 19308450 10.1007/s00436-009-1408-y

[brv70017-bib-0044] Bebber, D. P. , Ramotowski, M. A. T. & Gurr, S. J. (2013). Crop pests and pathogens move polewards in a warming world. Nature Climate Change 3(11), 985–988.

[brv70017-bib-0045] Beebe, W. (1951). Migration of insects (other than Lepidoptera) through Portachuelo Pass, Rancho Grande, north‐central Venezuela. Zoologica: Scientific Contributions of the New York Zoological Society 36(20), 255–266.

[brv70017-bib-0046] * Bello, F. J. , Segura, N. A. & Ruiz‐García, M. (2014). Analysis of the genetic variability and structure of *Ochlerotatus taeniorhynchus* (Diptera: Culicidae) populations from the Colombian Atlantic coast on the basis of random amplified polymorphic DNA markers. Genetics and Molecular Research 13(2), 4110–4123. 24938703 10.4238/2014.May.30.6

[brv70017-bib-0047] * Beppu, K. (1993). Seasonal migrations and age structure of adults of two dryomyzid species (Diptera, Dryomyzidae) in central Japan. Japanese Journal of Entomology 61(1), 23–30.

[brv70017-bib-0048] Beresford, D. V. & Sutcliffe, J. F. (2009). Local infestation or long‐distance migration? The seasonal recolonization of dairy farms by *Stomoxys calcitrans* (Diptera: Muscidae) in south Central Ontario, Canada. Journal of Economic Entomology 102(2), 788–798. 19449662 10.1603/029.102.0241

[brv70017-bib-0049] Biesmeijer, J. C. , Roberts, S. P. M. , Reemer, M. , Ohlemuller, R. , Edwards, M. , Peeters, T. , Schaffers, A. P. , Potts, S. G. , Kleukers, R. & Thomas, C. D. (2006). Parallel declines in pollinators and insect‐pollinated plants in Britain and The Netherlands. Science 313(5785), 351–354. 10.1126/science.1127863. 16857940

[brv70017-bib-0050] * Birke, A. , Guillén, L. , Midgarden, D. & Aluja, M. (2013). Fruit flies, *Anastrepha ludens* (Loew), *A. obliqua* (Macquart) and *A. grandis* (Macquart)(Diptera: Tephritidae): three pestiferous tropical fruit flies that could potentially expand their range to temperate areas. In Potential Invasive Pests, pp. 192–213, CAB International, Wall Ingford, UK.

[brv70017-bib-0051] Bishopp, F. C. (1913). The stable fly (*Stomoxys calcitrans* L.), an important live stock pest. Journal of Economic Entomology 6(1), 112–126.

[brv70017-bib-0052] * Bohart, G. E. & Nye, W. P. (1960). Insect pollinators of carrots in Utah. Utah Agricultural Experiment Station Bulletin 419, 1–16.

[brv70017-bib-0053] * Borkent, A. & Spinelli, G. R. (2007). Neotropical Ceratopogonidae (Diptera: Insecta), Edition (Volume 4). Pensoft Publishers, Sofia, Bulgaria.

[brv70017-bib-0054] * Boucher, M. , Collins, R. , Hesler, S. , Cox, K. & Loeb, G. (2021). The effect of *Erwinia amylovora* infection in apple saplings and fruit on the behavior of *Delia platura* (Diptera: Anthomyiidae). Environmental Entomology 50(1), 117–125. 33290563 10.1093/ee/nvaa153

[brv70017-bib-0055] * Boyd, A. M. & Weinstein, P. (1996). *Anopheles annulipes* Walker sl (Diptera: Culicidae), an under‐rated temperate climate malaria vector? New Zealand Entomologist 19(1), 35–41. 10.1080/00779962.1996.9722019.

[brv70017-bib-0056] * Brake, I. & Bächli, G. (2013). Drosophilidae (Diptera), Edition (Volume 9). Brill, Leiden, Netherlands.

[brv70017-bib-0057] * Braun, S. E. , Sanderson, J. P. & Wraight, S. P. (2012). Larval *Bradysia impatiens* (Diptera: Sciaridae) potential for vectoring *Pythium* root rot pathogens. Phytopathology 102(3), 283–289. 10.1094/PHYTO-09-11-0262. 22085299

[brv70017-bib-0058] Braverman, I. v. & Chechik, P. (1993). Introduction of Culicoides (Diptera, Ceratopogonidae). Israel Journal of Veterinary Medicine 48, 1–9.

[brv70017-bib-0059] * Braverman, Y. (1994). Nematocera (Ceratopogonidae, Psychodidae, Simuliidae, and Culicidae) and control methods. Revue scientifique et technique. Office international des épizooties 13(4), 1175–1199. 10.20506/rst.13.4.819 7711309

[brv70017-bib-0060] Brenton, L. C. L. (1844). The Septuagint Version of the Old Testament, According to the Vatican Text, Tr. Into English: with the Principal Various Readings of the Alexandrine Copy, and a Table of Comparative Chronology, Edition (Volume 1). S. Bagster, London, UK.

[brv70017-bib-0061] Brightwell, R. , Dransfield, R. D. , Stevenson, P. & Williams, B. (1997). Changes over twelve years in populations of *Glossina pallidipes* and *Glossina longipennis* (Diptera: Glossinidae) subject to varying trapping pressure at Nguruman, south‐west Kenya. Bulletin of Entomological Research 87(4), 349–370. 10.1017/S0007485300037378.

[brv70017-bib-0062] Bueno‐Marí, R. , Drago, A. , Montalvo, T. , Dutto, M. & Becker, N. (2022). Classic and novel tools for mosquito control worldwide. Ecology and Control of Vector‐borne Diseases 7, 234–238.

[brv70017-bib-0063] * Burmann, K. (1977). Syrphid migrations in mountainous regions: observations from Berichte des Naturwissenschaftlich‐medizinischen. In Vereins in Innsbruck (Volume 65), p. 129, Universitätsverlag Wagner, Innsbruck, Austria.

[brv70017-bib-0064] * Burton, J. F. (1990). Westerly autumn movement of *Eristalis* spec, near Heidelberg, south‐west Germany (Diptera, Syrphidae). Atalanta 21, 179–180.

[brv70017-bib-0065] * Butterworth, N. J. , Wallman, J. F. , Johnston, N. P. , Dawson, B. M. , Sharp‐Heward, J. & McGaughran, A. (2023). The blowfly *Chrysomya latifrons* inhabits fragmented rainforests, but shows no population structure. Oecologia 201(3), 703–719. 36773072 10.1007/s00442-023-05333-w PMC10038970

[brv70017-bib-0066] * Buxton, P. A. (1924). Applied entomology of Palestine, being a report to the Palestine Government. Bulletin of Entomological Research 14(3), 289–340.

[brv70017-bib-0067] * Cameron, E. C. , Sved, J. A. & Gilchrist, A. S. (2010). Pest fruit fly (Diptera: Tephritidae) in northwestern Australia: one species or two? Bulletin of Entomological Research 100(2), 197–206. 19602297 10.1017/S0007485309990150

[brv70017-bib-0068] Campbell, J. B. , Boxler, D. J. & Adams, D. C. (2002). Stable fly, *Stomoxys calcitrans*, (Diptera: Muscidae) numbers trapped at Nebraska sandhill pasture sites from 1998–2002. *The 2002 ESA Annual Meeting and Exhibition*.

[brv70017-bib-0069] * Carson, H. L. , Hardy, D. E. , Spieth, H. T. & Stone, W. S. (1970). The evolutionary biology of the Hawaiian Drosophilidae. Essays in Evolution and Genetics in Honor of Theodosius Dobzhansky: A Supplement to Evolutionary Biology 1, 437–543.

[brv70017-bib-0070] * Carvalho, S. D. & Kratz, F. L. (1988). Dispersão ativa em *Drosophila melanogaster* (Diptera; Drosophilidae). Revista Brasileira de Zoologia 5, 31–44.

[brv70017-bib-0071] * Castro, C. P. , Szpila, K. , Martínez‐Sánchez, A. , Rego, C. , Silva, I. , Serrano, A. R. & Boieiro, M. (2016). The blowflies of the Madeira archipelago: species diversity, distribution and identification (Diptera, Calliphoridaes. L.). ZooKeys 634(634), 101–123. 10.3897/zookeys.634.9262 PMC5126533 27917052

[brv70017-bib-0072] * Cerretti, P. , Stireman, J. O. III , Badano, D. , Gisondi, S. , Rognes, K. , Giudice, G. L. & Pape, T. (2019). Reclustering the cluster flies (Diptera: Oestroidea, Polleniidae). Systematic Entomology 44(4), 957–972.

[brv70017-bib-0073] * Champagne‐Cauchon, W. , Guay, J. F. , Fournier, V. & Cloutier, C. (2020). Phenology and spatial distribution of spotted‐wing drosophila (Diptera: Drosophilidae) in lowbush blueberry (Ericaceae) in Saguenay‐lac‐saint‐Jean, Québec, Canada. The Canadian Entomologist 152(4), 432–449. 10.4039/tce.2020.30.

[brv70017-bib-0074] * Chang, H. , Hsu, T. & Wu, W. (2001). Species diversity and seasonal fluctuation of fruit flies (Diptera: Tephritidae) in bamboo stands in Taipei. Formosan Entomologist 21(1), 47–64.

[brv70017-bib-0075] Chapman, J. W. , Nesbit, R. L. , Burgin, L. E. , Reynolds, D. R. , Smith, A. D. , Middleton, D. R. & Hill, J. K. (2010). Flight orientation behaviors promote optimal migration trajectories in high‐flying insects. Science 327(5966), 682–685. 20133570 10.1126/science.1182990

[brv70017-bib-0076] Chapman, J. W. , Nilsson, C. , Lim, K. S. , Bäckman, J. , Reynolds, D. R. & Alerstam, T. (2016). Adaptive strategies in nocturnally migrating insects and songbirds: contrasting responses to wind. Journal of Animal Ecology 85(1), 115–124. 26147535 10.1111/1365-2656.12420

[brv70017-bib-0077] Chapman, J. W. , Reynolds, D. R. , Smith, A. D. , Smith, E. T. & Woiwod, I. P. (2004). An aerial netting study of insects migrating at high altitude over England. Bulletin of Entomological Research 94(2), 123–136. 10.1079/ber2004287. 15153295

[brv70017-bib-0078] Chapman, J. W. , Reynolds, D. R. & Wilson, K. (2015). Long‐range seasonal migration in insects: mechanisms, evolutionary drivers and ecological consequences. Ecology Letters 18(3), 287–302. 25611117 10.1111/ele.12407

[brv70017-bib-0079] * Cherairia, M. & Adler, P. H. (2018). Genetic variation in a colonization specialist, *Simulium ruficorne* (Diptera: Simuliidae), the world's most widely distributed black fly. PLoS One 13(10), e0205137. 30281665 10.1371/journal.pone.0205137 PMC6169971

[brv70017-bib-0080] * Chin, H. C. , Kurahashi, H. , Marwi, M. A. , Jeffery, J. & Omar, B. (2011). Opportunistic insects associated with pig carrions in Malaysia. Sains Malaysiana 40(6), 601–604.

[brv70017-bib-0081] Chowdhury, S. , Fuller, R. A. , Dingle, H. , Chapman, J. W. & Zalucki, M. P. (2021). Migration in butterflies: a global overview. Biological Reviews 96(4), 1462–1483. 33783119 10.1111/brv.12714

[brv70017-bib-0082] Chown, S. L. & Language, K. (1994). Recently established Diptera and lepidoptera on sub‐antarctic Marion Island. African Entomology 2(1), 57–60.

[brv70017-bib-0083] * Cicero, J. M. , Adair, M. M. , Adair, R. C. , Hunter, W. B. , Avery, P. B. & Mizell, R. F. (2017). Predatory behavior of long‐legged flies (Diptera: Dolichopodidae) and their potential negative effects on the parasitoid biological control agent of the Asian citrus psyllid (Hemiptera: Liviidae). Florida Entomologist 100(2), 485–487. 10.1653/024.100.0243.

[brv70017-bib-0084] Čičková, H. , Pastor, B. , Kozánek, M. , Martínez‐Sánchez, A. , Rojo, S. & Takáč, P. (2012). Biodegradation of pig manure by the housefly, *Musca domestica*: a viable ecological strategy for pig manure management. PLoS One 7(3), e32798. 22431982 10.1371/journal.pone.0032798 PMC3303781

[brv70017-bib-0085] Clem, C. S. , Hobson, K. A. & Harmon‐Threatt, A. N. (2023). Insights into natal origins of migratory Nearctic hover flies (Diptera: Syrphidae): new evidence from stable isotope (δ2H) assignment analyses. Ecography 2023(2), e06465.

[brv70017-bib-0086] Colón‐González, F. J. , Sewe, M. O. , Tompkins, A. M. , Sjödin, H. , Casallas, A. , Rocklöv, J. , Caminade, C. & Lowe, R. (2021). Projecting the risk of mosquito‐borne diseases in a warmer and more populated world: a multi‐model, multi‐scenario intercomparison modelling study. The Lancet Planetary Health 5(7), e404–e414. 34245711 10.1016/S2542-5196(21)00132-7 PMC8280459

[brv70017-bib-0087] * Colwell, D. D. (2001). Bot flies and warble flies (order Diptera: family Oestridae). Parasitic diseases of wild mammals 2, 46–71.

[brv70017-bib-0088] * Cook, D. F. , Voss, S. C. , Finch, J. T. , Rader, R. C. , Cook, J. M. & Spurr, C. J. (2020). The role of flies as pollinators of horticultural crops: an Australian case study with worldwide relevance. Insects 11(6), 341. 10.3390/insects11060341. 32498457 PMC7349676

[brv70017-bib-0089] Cooke, S. J. , Piczak, M. L. , Singh, N. J. , Åkesson, S. , Ford, A. T. , Chowdhury, S. , Mitchell, G. W. , Norris, D. R. , Hardesty‐Moore, M. , McCauley, D. & Hammerschlag, N. (2024). Animal migration in the Anthropocene: threats and mitigation options. Biological Reviews 99(4), 1242–1260. 10.1111/brv.13066. 38437713

[brv70017-bib-0090] Costa‐Júnior, L. M. , Chaves, D. P. , Brito, D. R. B. , dos Santos, V. A. F. , Costa‐Júnior, H. N. & Barros, A. T. M. (2019). A review on the occurrence of *Cochliomyia hominivorax* (Diptera: Calliphoridae) in Brazil. Revista Brasileira de Parasitologia Veterinária 28(4), 548–562. 10.1590/s1984-29612019059. 31483031

[brv70017-bib-0091] Courtney, G. W. , Pape, T. , Skevington, J. H. & Sinclair, B. J. (2009). Biodiversity of diptera. Insect Biodiversity, 229–278. John Wiley & Sons, Chichester, UK.

[brv70017-bib-0092] * Coyne, J. A. & Milstead, B. (1987). Long‐distance migration of Drosophila. 3. Dispersal of *D. melanogaste*r alleles from a Maryland orchard. The American Naturalist 130(1), 70–82.

[brv70017-bib-0093] * Cupp, E. W. , Cupp, M. S. , Ribeiro, J. M. C. & Kunz, S. E. (1998). Blood‐feeding strategy of Haematobia irritans (Diptera: Muscidae). Journal of Medical Entomology 35(4), 591–595. 9701950 10.1093/jmedent/35.4.591

[brv70017-bib-0094] * Dällenbach, L. J. , Glauser, A. , Lim, K. S. , Chapman, J. W. & Menz, M. H. M. (2018). Higher flight activity in the offspring of migrants compared to residents in a migratory insect. Proceedings of the Royal Society B 285(1881), 20172829. 29925611 10.1098/rspb.2017.2829 PMC6030531

[brv70017-bib-0095] * Dalmat, H. T. (1955). The black flies Diptera, Simuliidae of Guatemala and their role as vectors of onchocerciasis. Smithsonian Miscellaneous Collections 125(1), 1–425.

[brv70017-bib-0096] * Danthanarayana, W. (1986). Lunar periodicity of insect flight and migration. In Insect Flight: Dispersal and Migration, pp. 88–119. Springer Berlin Heidelberg, Berlin, Heidelberg.

[brv70017-bib-0097] Dao, A. , Yaro, A. S. , Diallo, M. , Timbiné, S. , Huestis, D. L. , Kassogué, Y. , Traoré, A. I. , Sanogo, Z. L. , Samaké, D. & Lehmann, T. (2014). Signatures of aestivation and migration in Sahelian malaria mosquito populations. Nature 516(7531), 387–390. 25470038 10.1038/nature13987 PMC4306333

[brv70017-bib-0098] Davis, D. J. (2013). The phylogenetics of Tachinidae (Insecta: Diptera) with an emphasis on subfamily structure. (Doctoral dissertation, Wright State University).

[brv70017-bib-0099] * de Goeldlin Tiefenau, P. (1989). Note faunistique sur les Sphaerophoria (Diptères, Syrphidae) du Valais. Bulletin de la Murithienne 107, 35–46.

[brv70017-bib-0100] * De Lopes, O. S. , De Saccheta, L. A. , Francy, D. B. , Jakob, W. L. & Calisher, C. H. (1981). Emergence of a new arbovirus disease in Brazil: III. Isolation of Rocio virus from Psorophora ferox (Humboldt, 1819). American Journal of Epidemiology 113(2), 122–125. 6110335 10.1093/oxfordjournals.aje.a113075

[brv70017-bib-0101] * Delatte, H. , De Meyer, M. & Virgilio, M. (2019). Genetic structure and range expansion of *Zeugodacus* Cucurbitae (Diptera: Tephritidae) in Africa. Bulletin of Entomological Research 109(6), 713–722. 30724141 10.1017/S0007485319000026

[brv70017-bib-0102] * Deschepper, P. , Todd, T. N. , Virgilio, M. , De Meyer, M. , Barr, N. B. & Ruiz‐Arce, R. (2021). Looking at the big picture: worldwide population structure and range expansion of the cosmopolitan pest *Ceratitis capitata* (Diptera, Tephritidae). Biological Invasions 23, 3529–3543.

[brv70017-bib-0103] * Deutsch, F. & Kiss, B. (2021). Seasonal abundance changes of spotted wing drosophila in neighbouring habitats in Hungary. Proceedings 68, 1–6.

[brv70017-bib-0104] * Deyrup, M. & Deyrup, L. (2008). Flower visitation by adult shore flies at an inland site in Florida (Diptera: Ephydridae). Florida Entomologist 91(3), 504–507.

[brv70017-bib-0105] Dhimal, M. , Kramer, I. M. , Phuyal, P. , Budhathoki, S. S. , Hartke, J. , Ahrens, B. , Kuch, U. , Groneberg, D. A. , Nepal, S. & Liu, Q.‐Y. (2021). Climate change and its association with the expansion of vectors and vector‐borne diseases in the Hindu Kush Himalayan region: a systematic synthesis of the literature. Advances in Climate Change Research 12(3), 421–429. 10.1016/j.accre.2021.05.003.

[brv70017-bib-0106] Diakova, A. v. , Schepetov, D. M. , Oyun, N. Y. , Shatalkin, A. I. & Galinskaya, T. v. (2018). Assessing genetic and morphological variation in populations of Eastern European *Lucilia sericata* (Diptera: Calliphoridae). European Journal of Entomology 115, 192–197.

[brv70017-bib-0107] * Ding, J. T. , Adil, S. , Zhu, H. F. , Yu, F. & Alimasiand Luo, L. (2014). Flight capacity of adults of the ber fruit fly, *Carpomya vesuviana* (Diptera: Tephritidae). Acta Entomologica Sinica 57(11), 1315–1320.

[brv70017-bib-0108] Dingle, H. (2014). Migration: The Biology of Life on the Move. Oxford University Press, USA.

[brv70017-bib-0109] * Disney, R. H. L. & Disney, R. H. L. (1994). Scuttle flies: the Phoridae. In Adult Natural History, pp. 116–169, Springer Netherlands, Heidelberg, Germany.

[brv70017-bib-0110] Doyle, T. , Hawkes, W. L. S. , Massy, R. , Powney, G. D. , Menz, M. H. M. & Wotton, K. R. (2020). Pollination by hoverflies in the Anthropocene: pollination by hoverflies. Proceedings of the Royal Society B: Biological Sciences 287, 1–9. 10.1098/rspb.2020.0508 PMC7287354 32429807

[brv70017-bib-0111] Doyle, T. , Jimenez‐Guri, E. , Hawkes, W. L. S. , Massy, R. , Mantica, F. , Permanyer, J. , Cozzuto, L. , Hermoso Pulido, T. , Baril, T. , Hayward, A. & Wotton, K. R. (2022). Genome‐wide transcriptomic changes reveal the genetic pathways involved in insect migration. Molecular Ecology 31(16), 4332–4350. 10.1111/mec.16588. 35801824 PMC9546057

[brv70017-bib-0112] Doyle, T. , Poole, O. , Barnes, J. , Hawkes, W. L. S. , Jimenez Guri, E. & Wotton, K. R. (2025). Multiple factors contribute to female dominance in migratory bioflows. Open Biology 15(2), 240235. 10.1098/rsob.240235. 39933573 PMC11813574

[brv70017-bib-0113] * Drew, R. A. I. (2004). Biogeography and speciation in the Dacini (Diptera: Tephritidae: Dacinae). Bishop Museum Bulletin in Entomology 12, 165–178.

[brv70017-bib-0114] * Drew, R. A. I. , Zalucki, M. P. & Hooper, G. H. S. (1984). Ecological studies of Eastern Australian fruit flies (Diptera: Tephritidae) in their endemic habitat: I. Temporal variation in abundance. Oecologia 64, 267–272. 28312349 10.1007/BF00376881

[brv70017-bib-0115] * Ducheyne, E. , De ken, R. , Bécu, S. , Codina, B. , Nomikou, K. , Mangana‐Vougiaki, O. , Georgiev, G. , Purse, B. V. & Hendrick, G. (2007). Quantifying the wind dispersal of Culicoides species in Greece and Bulgaria. Geospatial Health 1(2), 177–189. 10.4081/gh.2007.266. 18686243

[brv70017-bib-0116] * Dziock, F. (2005). Evolution of prey specialization in aphidophagous syrphids of the genera *Melanostoma* and *Platycheirus* (Diptera: Syrphidae) 1. Body size, development and prey traits. European Journal of Entomology 102(3), 413–421.

[brv70017-bib-0117] * Eagles, D. , Deveson, T. , Walker, P. J. , Zalucki, M. P. & Durr, P. (2012). Evaluation of long‐distance dispersal of Culicoides midges into northern Australia using a migration model. Medical and Veterinary Entomology 26(3), 334–340. 22211884 10.1111/j.1365-2915.2011.01005.x

[brv70017-bib-0118] Eagles, D. , Melville, L. , Weir, R. , Davis, S. , Bellis, G. , Zalucki, M. P. , Walker, P. J. & Durr, P. A. (2014). Long‐distance aerial dispersal modelling of Culicoides biting midges: case studies of incursions into Australia. BMC Veterinary Research 10(1), 1–10. 24943652 10.1186/1746-6148-10-135 PMC4074460

[brv70017-bib-0119] Ebejer, M. J. & Bensusan, K. (2010). Hoverflies (Diptera, Syrphidae) recently encountered on Gibraltar, with two species new for Iberia. Dipterists Digest 17, 123–139.

[brv70017-bib-0120] Ecodiptera (2009). Ecodiptera – implementation of a management model for the ecologically sustainable treatment of pig manure in the Region of Los Serranos, Valencia‐Spain. https://tinyurl.com/y2qg4e3g.

[brv70017-bib-0121] * Edwards, J. S. (1972). Arthropod fallout on Alaskan snow. Arctic and Alpine Research 4(2), 167–176.

[brv70017-bib-0122] * Egedegbe, A. O. , Ojianwuna, C. C. , Enwemiwe, V. N. , Omotayo, A. I. , Eyeboka, D. N. & Esiwo, E. (2023). Molecular characterization and potentiality of Anopheles coluzzii in disease transmission in different communities in Ughelli North lga, Delta State, Nigeria. Animal Research International 20(2), 4988–5006.

[brv70017-bib-0123] Elser, J. J. , Fagan, W. F. , Denno, R. F. , Dobberfuhl, D. R. , Folarin, A. , Huberty, A. , Interlandi, S. , Kilham, S. S. , McCauley, E. & Schulz, K. L. (2000). Nutritional constraints in terrestrial and freshwater food webs. Nature 408(6812), 578–580. 10.1038/35046058. 11117743

[brv70017-bib-0124] El‐Wakeil, N. & Volkmar, C. (2011). Effect of weather conditions on frit fly (*Oscinella frit*, Diptera: Chloropidae) activity and infestation levels in spring wheat in central Germany. Gesunde Pflanzen 63(4), 159–165.

[brv70017-bib-0125] * Faiman, R. , Diallo, M. , Dao, A. , Djibril, S. , Sanogo, Z. L. , Sullivan, M. , Krishna, A. , Krajacich, B. J. & Lehmann, T. (2020). Quantifying flight aptitude variation in wild *Anopheles gambiae* in order to identify long‐distance migrants. Malaria Journal 19(1), 42005. 10.1186/s12936-020-03333-2 PMC7374819 32698842

[brv70017-bib-0126] * Farrow, R. A. (1984). Detection of transoceanic migration of insects to a remote Island in the Coral Sea, Willis Island. Australian Journal of Ecology 9(3), 253–272. 10.1111/j.1442-9993.1984.tb01362.x.

[brv70017-bib-0127] * Farwig, N. , Brandl, R. , Siemann, S. , Wiener, F. & Müller, J. (2014). Decomposition rate of carrion is dependent on composition not abundance of the assemblages of insect scavengers. Oecologia 175, 1291–1300. 24859425 10.1007/s00442-014-2974-y

[brv70017-bib-0128] * Feehan, J. , Hughes, R. D. , Bryce, M. A. & Runko, S. (1985). Bush fly abundance and population events in relation to dung beetle catches on the south coast of New South Wales. Australian Journal of Entomology 24(1), 37–43. 10.1111/j.1440-6055.1985.tb00182.x.

[brv70017-bib-0129] * Feltwell, J. (1976). Migration of *Hipparchia Semele* L. Journal of Research on Lepidoptera 15(2), 83–91.

[brv70017-bib-0130] * Ferenc, M. (2022). Mass swarm of a grass fly in Keszthely, Hungary. Journal of Central European Agriculture 23(4), 795–799.

[brv70017-bib-0131] * Figueiró, R. & Gil‐Azevedo, L. H. (2010). The role of neotropical blackflies (Diptera: Simuliidae) as vectors on the onchocerciasis: a short overview of the ecology behind the disease. Oecologia Australis 14(3), 745–755.

[brv70017-bib-0132] Finch, J. T. D. & Cook, J. M. (2020). Flies on vacation: evidence for the migration of Australian Syrphidae (Diptera). Ecological Entomology 45(4), 896–900.

[brv70017-bib-0133] Fischer, O. A. , Matlova, L. , Dvorska, L. , Švástová, P. , Bartoš, M. , Weston, R. T. & Pavlik, I. (2006). Various stages in the life cycle of syrphid flies (*Eristalis tenax*; Diptera: Syrphidae) as potential mechanical vectors of pathogens causing mycobacterial infections in pig herds. Folia Microbiologica 51(2), 147–153. 16821726 10.1007/BF02932171

[brv70017-bib-0134] Fisler, L. & Marcacci, G. (2022). Tens of thousands of migrating hoverflies found dead on a strandline in the South of France. Insect Conservation and Diversity 2022, 1–7.

[brv70017-bib-0135] Fleming, T. H. , Eby, P. , Kunz, T. H. & Fenton, M. B. (2003). Ecology of bat migration. Bat Ecology 156, 164–165.

[brv70017-bib-0136] * Fletcher, B. S. (1973). Observations on a movement of insects at Heron Island, Queensland. Australian Journal of Entomology 12(2), 157–160.

[brv70017-bib-0137] Florio, J. , Verú, L. M. , Dao, A. , Yaro, A. S. , Diallo, M. , Sanogo, Z. L. , Samaké, D. , Huestis, D. L. , Yossi, O. & Talamas, E. (2020). Diversity, dynamics, direction, and magnitude of high‐altitude migrating insects in the Sahel. Scientific Reports 10(1), 1–14. 33239619 10.1038/s41598-020-77196-7 PMC7688652

[brv70017-bib-0138] * Foil, L. D. (1989). Tabanids as vectors of disease agents. Parasitology Today 5(3), 88–96. 15463186 10.1016/0169-4758(89)90009-4

[brv70017-bib-0139] Francuski, L. , Djurakic, M. , Ludoški, J. , Hurtado, P. , Pérez‐Bañón, C. , Ståhls, G. , Rojo, S. & Milankov, V. (2014). Shift in phenotypic variation coupled with rapid loss of genetic diversity in captive populations of *Eristalis tenax* (Diptera: Syrphidae): consequences for rearing and potential commercial use. Journal of Economic Entomology 107(2), 821–832. 24772566 10.1603/ec13243

[brv70017-bib-0140] * Francuski, L. , Djurakic, M. , Ludoški, J. & Milankov, V. (2013). Landscape genetics and spatial pattern of phenotypic variation of *Eristalis tenax* across Europe. Journal of Zoological Systematics and Evolutionary Research 51(3), 227–238. 10.1111/jzs.12017.

[brv70017-bib-0141] Funk, C. , Hoell, A. , Shukla, S. , Husak, G. & Michaelsen, J. (2016). The East African monsoon system: seasonal climatologies and recent variations. In The Monsoons and Climate Change: Observations and Modeling, pp. 163–185, Springer, Heidelberg, Germany.

[brv70017-bib-0142] * Galante, E. & Marcos‐Garcia, M. A. (2008). Decomposer insects. In Encyclopedia of Entomology, pp. 1158–1169, Centro Iberoamericano de la Biodiversidad, Alicante, Spain.

[brv70017-bib-0143] Gao, B. , Wotton, K. R. , Hawkes, W. L. S. , Menz, M. H. M. , Reynolds, D. R. , Zhai, B.‐P. , Hu, G. & Chapman, J. W. (2020 *a*). Adaptive strategies of high‐flying migratory hoverflies in response to wind currents. Proceedings of the Royal Society B 287(1928), 20200406. 32486972 10.1098/rspb.2020.0406 PMC7341907

[brv70017-bib-0144] * Gao, J. , Zhang, H. , Guo, X. , Xing, D. , Dong, Y. , Lan, C. , Wang, G. , Li, C. & Zhao, T. (2020b). *Temporal genetic variation and dispersal patterns of* Aedes albopictus *(Diptera: Culicidae) among three different temperature regions of China* . Doctoral dissertation: Institute of Microbiology.

[brv70017-bib-0145] * Garden, A. & Davies, R. W. (1988). Decay rates of autumn and spring leaf litter in a stream and effects on growth of a detritivore. Freshwater Biology 19(3), 297–303.

[brv70017-bib-0146] Garms, R. , Walsh, J. F. & Davies, J. B. (1979). Studies on the reinvasion of the Onchocerciasis Control Programme in the Volta River Basin by *Simulium damnosum* sI with emphasis on the south‐western areas. Tropenmedizin und Parasitologie 30(3), 345–362. 575581

[brv70017-bib-0147] Gatter, W. (1977). Eine Wanderung der Erdschnake (*Tipula oleracea* l.). Passive Verdriftung oder gerichtete Migration. Nachrichtenblatt Bayerischer Entomologen 26, 141–152.

[brv70017-bib-0148] Gatter, W. (1980). Nordwärts gerichtete Frühjahrswanderungen palaearktischer Schmetterlinge, Fliegen und Hummeln im Himalaya‐und Transhimalayagebiet Nepals. Atalanta 11, 188–196.

[brv70017-bib-0149] Gatter, W. , Ebenhöh, H. , Kima, R. , Gatter, W. & Scherer, F. (2020). 50‐jährige Untersuchungen an migrierenden Schwebfliegen, Waffenfliegen und Schlupfwespen belegen extreme Rückgänge (Diptera: Syrphidae, Stratiomyidae; Hymenoptera: Ichneumonidae). Entomologische Zeitschrift Schwanfeld 130(3), 131–142.

[brv70017-bib-0150] * Gatter, W. & Schmid, U. (1990). Hoverfly migration (Diptera Syrphidae) at Randecker maar southwest Germany. Spixiana Supplement 15, 1–100.

[brv70017-bib-0151] * Gendernalik, A. , Weger‐Lucarelli, J. , Luna, S. M. G. , Fauver, J. R. , Rückert, C. , Murrieta, R. A. , Bergren, N. , Samaras, D. , Nguyen, C. , Kading, R. C. & Ebel, G. D. (2017). American *Aedes vexans* mosquitoes are competent vectors of Zika virus. The American Journal of Tropical Medicine and Hygiene 96(6), 1338–1340. 10.4269/ajtmh.16-0963. 28719283 PMC5462567

[brv70017-bib-0152] Gerry, A. C. (2007). Predicting and Controlling Stable Flies on California Dairies. UCANR Publications, California.

[brv70017-bib-0153] Gibernau, M. , Macquart, D. , Diaz, A. , House, D. , Fern‐Barrow, P. & Dorset, B. (2003). Pollen viability and longevity in two species of Arum. Aroideana 26, 58–62.

[brv70017-bib-0154] * Gisondi, S. , Buenaventura, E. , Jensen, A. R. , Stireman, J. O. III , Nihei, S. S. , Pape, T. & Cerretti, P. (2023). Phylogenetic relationships of the woodlouse flies (Diptera: Rhinophorinae) and the cluster flies (Diptera: Polleniidae). PLoS One 18(9), e0285855. 37725599 10.1371/journal.pone.0285855 PMC10508628

[brv70017-bib-0155] Glick, P. A. (1939). The Distribution of Insects, Spiders, and Mites in the Air, Edition (Volume 673), pp. 1–151. United States Department of Agriculture Technical bulletin, Washington DC.

[brv70017-bib-0156] * Glick, P. A. (1960). Collecting insects by airplane, with special reference to dispersal of the potato leafhopper (No. 1222). US Department of Agriculture.

[brv70017-bib-0157] * González, R. , Wilkerson, R. , Fidel Suárez, M. , García, F. , Gallego, G. , Cárdenas, H. , Elisa Posso, C. & Cristina Duque, M. (2007). A population genetics study of *Anopheles darlingi* (Diptera: Culicidae) from Colombia based on random amplified polymorphic DNA‐polymerase chain reaction and amplified fragment length polymorphism markers. Memórias do Instituto Oswaldo Cruz 102, 255–262. 17568929 10.1590/s0074-02762007005000037

[brv70017-bib-0158] Goulson, D. (2019). The insect apocalypse, and why it matters. Current Biology 29(19), 967–971. 10.1016/j.cub.2019.06.069 31593678

[brv70017-bib-0159] * Graczyk, T. K. , Knight, R. , Gilman, R. H. & Cranfield, M. R. (2001). The role of non‐biting flies in the epidemiology of human infectious diseases. Microbes and Infection 3(3), 231–235. 11358717 10.1016/s1286-4579(01)01371-5

[brv70017-bib-0160] * Gratz, N. G. (2004). Critical review of the vector status of Aedes albopictus. Medical and Veterinary Entomology 18(3), 215–227. 15347388 10.1111/j.0269-283X.2004.00513.x

[brv70017-bib-0161] Greathead, D. J. (1962). The biology of *Stomorhina lunata* (Fabricius)(Diptera: Calliphoridae), a predator of the eggs of Acrididae. Proceedings of the Zoological Society of London 139(1), 139–180.

[brv70017-bib-0162] * Griffiths, G. C. (1986). Phenology and dispersion of *Delia radicum* (L.) (Diptera: Anthomyiidae) in canola fields at Morinville, Alberta. Quaestiones Entomologicae. 22(1), 2–50.

[brv70017-bib-0163] Guerra, P. A. , Gegear, R. J. & Reppert, S. M. (2014). A magnetic compass aids monarch butterfly migration. Nature Communications 5(1), 1–8. 10.1038/ncomms5164 PMC4090716 24960099

[brv70017-bib-0164] Guerra, P. C. , Keil, C. B. , Stevenson, P. C. , Mina, D. , Samaniego, S. , Peralta, E. , Mazon, N. & Chancellor, T. C. B. (2017). Larval performance and adult attraction of *Delia platura* (Diptera: Anthomyiidae) in a native and an introduced crop. Journal of Economic Entomology 110(1), 186–191. 10.1093/jee/tow237. 28011683

[brv70017-bib-0165] * Gunstream, S. E. & Chew, R. M. (1967). The ecology of *Psorophora confinnis* (Diptera: Culicidae) in southern California. II. Temperature and development. Annals of the Entomological Society of America 60(2), 434–439. 4382891 10.1093/aesa/60.2.434

[brv70017-bib-0166] Guo, J. , Fu, X. , Zhao, S. , Shen, X. , Wyckhuys, K. A. G. & Wu, K. (2020). Long‐term shifts in abundance of (migratory) crop‐feeding and beneficial insect species in northeastern Asia. Journal of Pest Science 93(2), 583–594.

[brv70017-bib-0167] * Gustani, E. C. , Oliveira, A. P. F. , Santos, M. H. , Machado, L. P. & Mateus, R. P. (2015). Demographic structure and evolutionary history of *Drosophila ornatifrons* (Diptera, Drosophilidae) from Atlantic Forest of Southern Brazil. Zoological Science 32(2), 141–150. 10.2108/zs140062. 25826062

[brv70017-bib-0168] Haest, B. , Liechti, F. , Hawkes, W. L. , Chapman, J. , Åkesson, S. , Shamoun‐Baranes, J. , Nesterova, A. P. , Comor, V. , Preatoni, D. & Bauer, S. (2024). Continental‐scale patterns in diel flight timing of high‐altitude migratory insects. Philosophical Transactions of the Royal Society B 379(1904), 20230116. 10.1098/rstb.2023.0116 PMC11070267 38705191

[brv70017-bib-0169] * Hall, D. R. , Amarawardana, L. , Cross, J. V. , Francke, W. , Boddum, T. & Hillbur, Y. (2012). The chemical ecology of cecidomyiid midges (Diptera: Cecidomyiidae). Journal of Chemical Ecology 38, 2–22. 22215563 10.1007/s10886-011-0053-y

[brv70017-bib-0170] Hallmann, C. A. , Ssymank, A. , Sorg, M. , de Kroon, H. & Jongejans, E. (2021). Insect biomass decline scaled to species diversity: general patterns derived from a hoverfly community. Proceedings of the National Academy of Sciences 118(2), e2002554117. 10.1073/pnas.2002554117 PMC7812780 33431568

[brv70017-bib-0171] Hallworth, M. T. , Marra, P. P. , McFarland, K. P. , Zahendra, S. & Studds, C. E. (2018). Tracking dragons: stable isotopes reveal the annual cycle of a long‐distance migratory insect. Biology Letters 14(12), 20180741. 10.1098/rsbl.2018.0741. 30958242 PMC6303508

[brv70017-bib-0172] * Hanson, S. M. , Novak, R. J. , Lampman, R. L. & Vodkin, M. H. (1995). Notes on the biology of Orthopodomyia in Illinois. Journal of the American Mosquito Control Association 11(3), 375–376. 8551313

[brv70017-bib-0173] Hardy, A. C. & Cheng, L. (1986). Studies in the distribution of insects by aerial currents. III. Insect drift over the sea. Ecological Entomology 11(3), 283–290.

[brv70017-bib-0174] * Hardy, A. C. & Milne, P. S. (1938). Studies in the distribution of insects by aerial currents. The Journal of Animal Ecology 7(2), 199–229. 10.2307/1156.

[brv70017-bib-0175] * Harris, E. J. , Takara, J. M. & Nishida, T. (1986). Distribution of the melon fly, *Dacus cucurbitae* (Diptera: Tephritidae), and host plants on Kauai, Hawaiian Islands. Environmental Entomology 15(3), 488–493. 10.1093/ee/15.3.488.

[brv70017-bib-0176] Hassall, C. , Owen, J. & Gilbert, F. (2017). Phenological shifts in hoverflies (Diptera: Syrphidae): linking measurement and mechanism. Ecography 40(7), 853–863.

[brv70017-bib-0177] Hawkes, W. L. , Doyle, T. , Massy, R. , Weston, S. T. , Davies, K. , Cornelius, E. , Collier, C. , Chapman, J. W. , Reynolds, D. R. & Wotton, K. R. (2024). The most remarkable migrants—systematic analysis of the Western European insect flyway at a Pyrenean mountain pass. Proceedings of the Royal Society B 291, 20232831. 38864145 10.1098/rspb.2023.2831 PMC11285860

[brv70017-bib-0178] Hawkes, W. L. , Walliker, E. , Gao, B. , Forster, O. , Lacey, K. , Doyle, T. , Massy, R. , Roberts, N. W. , Reynolds, D. R. & Özden, Ö. (2022a). Huge spring migrations of insects from the Middle East to Europe: quantifying the migratory assemblage and ecosystem services. Ecography 2022(10), e06288. 10.1111/ecog.06288.

[brv70017-bib-0179] Hawkes, W. L. , Weston, S. T. , Cook, H. , Doyle, T. , Massy, R. , Guri, E. J. , Wotton Jimenez, R. E. & Wotton, K. R. (2022 *b*). Migratory hoverflies orientate north during spring migration. Biology Letters 18(10), 20220318. 36196552 10.1098/rsbl.2022.0318 PMC9533008

[brv70017-bib-0180] * Heath, A. C. G. (1982). Beneficial aspects of blowflies (Diptera: Calliphoridae). New Zealand Entomologist 7(3), 343–348.

[brv70017-bib-0181] * Heiduk, A. , Kong, H. , Brake, I. , von Tschirnhaus, M. , Tolasch, T. , Tröger, A. G. , Wittenberg, E. , Francke, W. , Meve, U. & Dötterl, S. (2015). Deceptive *Ceropegia dolichophylla* fools its kleptoparasitic fly pollinators with exceptional floral scent. Frontiers in Ecology and Evolution 3, 66.

[brv70017-bib-0182] * Heiduk, A. , Meve, U. , Menzel, F. , Haenni, J. P. , Tschirnhaus, M. V. , Dötterl, S. & Johnson, S. D. (2021). Fly pollination of kettle trap flowers of *Riocreuxia torulosa* (Ceropegieae‐Anisotominae): a generalized system of floral deception. Plants 10(8), 1564. 34451609 10.3390/plants10081564 PMC8398993

[brv70017-bib-0183] Hemingway, J. , Penilla, R. P. , Rodriguez, A. D. , James, B. M. , Edge, W. , Rogers, H. & Rodriguez, M. H. (1997). Resistance management strategies in malaria vector mosquito control. A large‐scale field trial in Southern Mexico. Pesticide Science 51, 375–382. 10.1002/(sici)1096-9063(199711)51:3<>3.0.co;2-k.

[brv70017-bib-0184] * Hernández, M. C. (2008). Biology of Thrypticus truncatus and *Thrypticus sagittatus* (Diptera: Dolichopodidae), petiole miners of water hyacinth, in Argentina, with morphological descriptions of larvae and pupae. Annals of the Entomological Society of America 101(6), 1041–1049.

[brv70017-bib-0185] * Hewadikaram, K. A. & Goff, M. L. (1991). Effect of carcass size on rate of decomposition and arthropod succession patterns. The American Journal of Forensic Medicine and Pathology 12(3), 235–240. 1750396 10.1097/00000433-199109000-00013

[brv70017-bib-0186] Hill, L. (2013). Long‐term light trap data from Tasmania, Australia. Plant Protection Quarterly 28(1), 22–27.

[brv70017-bib-0187] * Hiwat, H. & Bretas, G. (2011). Ecology of *Anopheles darlingi* Root with respect to vector importance: a review. Parasites & Vectors 4, 1–13. 21923902 10.1186/1756-3305-4-177 PMC3183005

[brv70017-bib-0188] Hlaváček, A. , Lučan, R. K. & Hadrava, J. (2022). Autumnal migration patterns of hoverflies (Diptera: Syrphidae): interannual variability in timing and sex ratio. PeerJ 10, e14393. 10.7717/peerj.14393. 36523467 PMC9745789

[brv70017-bib-0189] Hobson, K. A. , Anderson, R. C. , Soto, D. X. & Wassenaar, L. I. (2012). Isotopic evidence that dragonflies (*Pantala flavescens*) migrating through the Maldives come from the Northern Indian subcontinent. PLoS One 7(12), e52594. 23285106 10.1371/journal.pone.0052594 PMC3527571

[brv70017-bib-0190] * Hoffmann, A. & Hering, D. (2000). Wood‐associated macroinvertebrate fauna in Central European streams. International Review of Hydrobiology: A Journal Covering all Aspects of Limnology and Marine Biology 85(1), 25–48.

[brv70017-bib-0191] Høgåsen, H. R. (1988). Physiological Changes Associated with the Diadromous Migration of Salmonids (no. 127). NRC Research Press, Ontario, Canada.

[brv70017-bib-0192] Hogsette, J. A. & Ruff, J. P. (1985). Stable fly (Diptera: Muscidae) migration in northwest Florida. Environmental Entomology 14(2), 170–175. 10.1093/ee/14.2.170.

[brv70017-bib-0193] * Hogsette, J. A. , Ruff, J. P. & Jones, C. J. (1989). Dispersal behavior of stable flies (Diptera: Muscidae). Miscellaneous Publications of the Entomological Society of America 74, 23–32.

[brv70017-bib-0194] * Holzapfel, E. P. & Harrell, J. C. (1968). Transoceanic dispersal studies of insects. Pacific Insects 10(1), 115–153.

[brv70017-bib-0195] * Hondelmann, P. , Borgemeister, C. & Poehling, H.‐M. (2005). Restriction fragment length polymorphisms of different DNA regions as genetic markers in the hoverfly *Episyrphus balteatus* (Diptera: Syrphidae). Bulletin of Entomological Research 95(4), 349–359. 16048683 10.1079/ber2005366

[brv70017-bib-0196] Hondelmann, P. & Poehling, H. (2007). Diapause and overwintering of the hoverfly *Episyrphus balteatus* . Entomologia Experimentalis et Applicata 124(2), 189–200. 10.1111/j.1570-7458.2007.00568.x.

[brv70017-bib-0197] Howard, E. & Davis, A. K. (2009). The fall migration flyways of monarch butterflies in eastern North America revealed by citizen scientists. Journal of Insect Conservation 13, 279–286.

[brv70017-bib-0198] Hu, G. , Feng, H. , Otuka, A. , Reynolds, D. R. , Drake, V. A. & Chapman, J. W. (2024). The East Asian Insect Flyway: geographical and climatic factors driving migration among diverse crop pests. Annual Review of Entomology 5(70), 1–22. 10.1146/annurev-ento-012524-124018 39499909

[brv70017-bib-0199] Hu, G. , Lim, K. S. , Horvitz, N. , Clark, S. J. , Reynolds, D. R. , Sapir, N. & Chapman, J. W. (2016). Mass seasonal bioflows of high‐flying insect migrants. Science 354(6319), 1584–1587. 28008067 10.1126/science.aah4379

[brv70017-bib-0200] Hu, G. , Stefanescu, C. , Oliver, T. H. , Roy, D. B. , Brereton, T. , van Swaay, C. , Reynolds, D. R. & Chapman, J. W. (2021). Environmental drivers of annual population fluctuations in a trans‐Saharan insect migrant. Proceedings of the National Academy of Sciences 118(26), e2102762118. 10.1073/pnas.2102762118 PMC8256005 34155114

[brv70017-bib-0201] * Hubenov, Z. (2008). Composition and zoogeographical characteristics of the family Tachinidae (Diptera: Insecta) in Serbia and Bulgaria. Advances in Arachnology and Developmental Biology 12, 375–394.

[brv70017-bib-0202] * Huerta, H. & Ibañez‐Bernal, S. (1998). First record of the genera *Nilobezzia Kieffer* and *Schizonyxhelea Clastrier* in Mexico (Diptera: Ceratopogonidae). Folia Entomológica Mexicana 102, 71–73.

[brv70017-bib-0203] Huestis, D. L. , Dao, A. , Diallo, M. , Sanogo, Z. L. , Samake, D. , Yaro, A. S. , Ousman, Y. , Linton, Y.‐M. , Krishna, A. & Veru, L. (2019). Windborne long‐distance migration of malaria mosquitoes in the Sahel. Nature 574(7778), 404–408. 10.1038/s41586-019-1622-4. 31578527 PMC11095661

[brv70017-bib-0204] * Hughes, P. S. (1975). The biology of *Archytas marmoratus* (Townsend). Annals of the Entomological Society of America 68(4), 759–767.

[brv70017-bib-0205] * Hui, Y. E. (2001). Distribution of the oriental fruit fly (Diptera: Tephritidae) in Yunnan Province. Insect Science 8(2), 175–182.

[brv70017-bib-0206] * Imada, Y. & Kato, M. (2016). Bryophyte‐feeders in a basal brachyceran lineage (Diptera: Rhagionidae: Spaniinae): adult oviposition behavior and changes in the larval mouthpart morphology accompanied with the diet shifts. PLoS One 11(11), e0165808. 27812169 10.1371/journal.pone.0165808 PMC5094795

[brv70017-bib-0207] * Iqbal, W. , Malik, M. F. , Sarwar, M. K. , Azam, I. , Iram, N. & Rashda, A. (2014). Role of housefly (*Musca domestica*, Diptera; Muscidae) as a disease vector; a review. Journal of Entomology and Zoology Studies 2(2), 159–163.

[brv70017-bib-0208] * Israely, N. , Ziv, Y. & Oman, S. D. (2005). Spatiotemporal distribution patterns of Mediterranean fruit fly (Diptera: Tephritidae) in the central region of Israel. Annals of the Entomological Society of America 98(1), 77–84.

[brv70017-bib-0209] * Iwasaki, A. , Miyake, N. , Takezawa, Y. , Mizukoshi, T. , Iwaizumi, R. & Uebori, T. (2008). Wind‐dependant spring migration of *Chromatomyia horticola* (Goureau)(Diptera: Agromyzidae) in Hokkaido, the northern Island of Japan. Japanese Journal of Applied Entomology and Zoology 52(3), 129–137.

[brv70017-bib-0210] Jacquard, C. , Virgilio, M. , David, P. , Quilici, S. , de Meyer, M. & Delatte, H. (2013). Population structure of the melon fly, *Bactrocera cucurbitae*, in Reunion Island. Biological Invasions 15, 759–773.

[brv70017-bib-0211] * Jeekel, C. A. W. & Overbeek, H. (1968). A migratory flight of hover‐flies (Diptera, Syrphidae) observed in Austria. Beaufortia 15(196), 123–126.

[brv70017-bib-0212] Jenni, L. & Schaub, M. (2003). Behavioural and physiological reactions to environmental variation in bird migration: a review. Avian migration 1, 155–171.

[brv70017-bib-0213] Jensen, J.‐K. (2001). An invasion of migrating insects (Syrphidae and Lepidoptera) on The Faroe Islands in September 2000. Norwegian Journal Of Entomology 48, 263–268.

[brv70017-bib-0214] Jia, H. , Liu, Y. , Li, X. , Li, H. , Pan, Y. , Hu, C. , Zhou, X. , Wyckhuys, K. A. G. & Wu, K. (2022). Windborne migration amplifies insect‐mediated pollination services. eLife 11, e76230. 10.7554/elife.76230. 35416148 PMC9042232

[brv70017-bib-0215] * Johansen, C. A. , Farrow, R. A. , Morrisen, A. , Foley, P. , Bellis, G. , Van Den Hurk, A. F. , Montgomery, B. , Mackenzie, J. S. & Ritchie, S. A. (2003). Collection of wind‐borne haematophagous insects in the Torres Strait, Australia. Medical and Veterinary Entomology 17(1), 102–111. 12680932 10.1046/j.1365-2915.2003.00413.x

[brv70017-bib-0216] Johnson, C. G. (1969). Migration and Dispersal of Insects by Flight. Methuen, London.

[brv70017-bib-0217] * Johnson, C. G. , Taylor, L. R. & Southwood, T. R. E. (1962). High altitude migration of *Oscinella fri*t L.(Diptera: Chloropidae). The Journal of Animal Ecology 31(2), 373–383. 10.2307/2148.

[brv70017-bib-0218] * Johnson, C. G. , Walsh, J. F. , Davies, J. B. , Clark, S. J. & Perry, J. N. (1985). The pattern and speed of displacement of females of *Simulium damnosum* Theobald sl (Diptera: Simuliidae) across the Onchocerciasis Control Programme area of West Africa in 1977 and 1978. Bulletin of Entomological Research 75(1), 73–92. 10.1017/S0007485300014188.

[brv70017-bib-0219] * Johnson, J. B. G. (1976). Migrating and other terrestrial insects at sea. Marine Insects, 97–117, American Elsevier, New York, USA.

[brv70017-bib-0220] * Jones, C. J. , Hogsette, J. A. , Patterson, R. S. , Milne, D. E. , Propp, G. D. , Milio, J. F. , Rickard, L. G. & Ruff, J. P. (1991). Origin of stable flies (Diptera: Muscidae) on west Florida beaches: electrophoretic analysis of dispersal. Journal of Medical Entomology 28(6), 787–795. 1770514 10.1093/jmedent/28.6.787

[brv70017-bib-0221] Jones, C. M. , Parry, H. , Tay, W. T. , Reynolds, D. R. & Chapman, J. W. (2019). Movement ecology of pest *Helicoverpa*: implications for ongoing spread. Annual Review of Entomology 64, 277–295. 10.1146/annurev-ento-011118-111959 30296859

[brv70017-bib-0222] * Jones, W. R. , Cochran, D. G. , Graham, P. P. & Kelly, R. F. (1973). Growth and development of the cheese skipper under sterile, unsterile, and sterile‐inoculated conditions. Journal of Economic Entomology 66(6), 1252–1254. 10.1093/jee/66.6.1252.

[brv70017-bib-0223] * Kahana‐Sutin, E. , Klement, E. , Lensky, I. & Gottlieb, Y. (2017). High relative abundance of the stable fly *Stomoxys calcitrans* is associated with lumpy skin disease outbreaks in Israeli dairy farms. Medical and Veterinary Entomology 31(2), 150–160. 27976815 10.1111/mve.12217

[brv70017-bib-0224] * Kameke, D. , Kampen, H. , Wacker, A. & Werner, D. (2021). Field studies on breeding sites of *Culicoides* Latreille (Diptera: Ceratopogonidae) in agriculturally used and natural habitats. Scientific Reports 11(1), 10007. 33976240 10.1038/s41598-021-86163-9 PMC8113236

[brv70017-bib-0225] * Kanmiya, K. (2002). Flight properties of orthorrhaphous brachycera flies in tethered flight performance (Insecta: Diptera). Medical Entomology and Zoology 53(Supplement2), 109–120. 10.7601/mez.53.109.

[brv70017-bib-0226] * Karpachkvsky, L. O. , Perel, T. S. & Bartsevich, V. V. (1968). The role of Bibionidae larvae in decomposition of forest litter. Pedobiologia 8(1), 146–149.

[brv70017-bib-0227] * Kastinger, C. & Weber, A. (2001). Bee‐flies (*Bombylius* spp., Bombyliidae, Diptera) and the pollination of flowers. Flora 196(1), 3–25.

[brv70017-bib-0228] * Kaufmann, T. (1975). Studies on the ecology and biology of a cocoa pollinator, *Forcipomyia squamipennis* I. & M. (Diptera, Ceratopogonidae), in Ghana. Bulletin of Entomological Research 65(2), 263–268.

[brv70017-bib-0229] * Kaushik, P. K. , Renz, M. O. & Shannon, B. (2020). Characterizing long‐range search behavior in Diptera using complex 3D virtual environments. Proceedings of the National Academy of Sciences 117(22), 12201–12207. 10.1073/pnas.1912124117 PMC7275712 32424090

[brv70017-bib-0230] * Kautz, A. R. & Gardiner, M. M. (2019). Agricultural intensification may create an attractive sink for Dolichopodidae, a ubiquitous but understudied predatory fly family. Journal of Insect Conservation 23, 453–465.

[brv70017-bib-0231] * Kay, B. H. & Farrow, R. A. (2000). Mosquito (Diptera: Culicidae) dispersal: implications for the epidemiology of Japanese and Murray Valley encephalitis viruses in Australia. Journal of Medical Entomology 37(6), 797–801. 10.1603/0022-2585-37.6.797. 11126532

[brv70017-bib-0232] Keaster, A. J. , Grundler, J. A. , Craig, W. S. & Jackson, M. A. (1996). Noctuid moths and other insects captured in wing‐style traps baited with black cutworm (Lepidoptera: Noctuidae) pheromone on offshore oil platforms in the Gulf of Mexico, 1988–1991. Journal of the Kansas Entomological Society 69(1), 17–25.

[brv70017-bib-0233] * Kehlmaier, C. (2002). Ein kleiner Beitrag zur Wanderaktivität von Schwebfliegen auf dem Mittelmecr (Diptera, Syrphidae). Wolucella 6, 154–156.

[brv70017-bib-0234] Kennedy, J. S. (1985). Migration, behavioral and ecological. In Migration: Mechanisms and Adaptive Significance, 27, University of Texas Press, USA.

[brv70017-bib-0235] * Kevan, P. G. & Baker, H. G. (1983). Insects as flower visitors and pollinators. Annual Review of Entomology 28(1), 407–453.

[brv70017-bib-0236] * Kim, Y. & Kim, D. S. (2016). Integrated pest management against Bactrocera fruit flies. Korean Journal of Applied Entomology 55(4), 359–376.

[brv70017-bib-0237] * Kimura, M. T. & Beppu, K. (1993). Climatic adaptations in the *Drosophila immigrans* species group: seasonal migration and thermal tolerance. Ecological Entomology 18(2), 141–149. 10.1111/j.1365-2311.1993.tb01195.x.

[brv70017-bib-0238] Kinney, K. K. , Lindroth, R. L. , Jung, S. M. & Nordheim, E. v. (1997). Effects of CO_2_ and NO_3_− availability on deciduous trees: phytochemistry and insect performance. Ecology 78(1), 215–230.

[brv70017-bib-0239] * Klimesova, V. , Oleksakova, T. , Barták, M. & Sulakova, H. (2016). Forensically important Muscidae (Diptera) associated with decomposition of carcasses and corpses in The Czech Republic. Mendel Net 23, 784–789.

[brv70017-bib-0240] Knoblauch, A. , Thoma, M. & Menz, M. H. M. (2021). Autumn southward migration of dragonflies along the Baltic coast and the influence of weather on flight behaviour. Animal Behaviour 176, 99–109.

[brv70017-bib-0241] * Knutson, L. & Vala, J. C. (2011). Biology of Snail‐Killing Sciomyzidae Flies. Cambridge University Press, Cambridge, UK.

[brv70017-bib-0242] * Kourti, A. (2004). Estimates of gene flow from rare alleles in natural populations of medfly *Ceratitis capitata* (Diptera: Tephritidae). Bulletin of Entomological Research 94(5), 449–456. 15385064 10.1079/ber2004324

[brv70017-bib-0243] * Krafsur, E. S. (2009). Tsetse flies: genetics, evolution, and role as vectors. Infection, Genetics and Evolution 9(1), 124–141. 10.1016/j.meegid.2008.09.010. PMC2652644 18992846

[brv70017-bib-0244] * Krandčmar, S. , Mikuska, A. & Merdić, E. (2006). Response of Tabanidae (Diptera) to different natural attractants. Journal of Vector Ecology 31(2), 262–265. 17249343 10.3376/1081-1710(2006)31[262:rotdtd]2.0.co;2

[brv70017-bib-0245] * Krčmar, S. , Kučinić, M. , Durbešić, P. & Benović, A. (2010). Insects from the middle of the Adriatic Sea. Entomologia Croatica 14(3–4), 75–84.

[brv70017-bib-0246] * Krinsky, W. L. (2019). Tsetse flies (Glossinidae). In Medical and Veterinary Entomology, pp. 369–382, Academic Press, London, UK.

[brv70017-bib-0247] * Krpač, V. (2021). Review on hoverflies (Diptera, Syrphidae) fauna in Sharr Mountains. Journal of Science, Environment and Technology‐ECOTEC 3(5–6), 38–149.

[brv70017-bib-0248] * Krüger, F. , Clare, E. L. , Symondson, W. O. , Keišs, O. & Pētersons, G. (2014). Diet of the insectivorous bat *Pipistrellus nathusii* during autumn migration and summer residence. Molecular Ecology 23(15), 3672–3683. 24118366 10.1111/mec.12547

[brv70017-bib-0249] * Kumar, S. , Joshi, P. C. , Nath, P. , Singh, V. K. & Mansotra, D. K. (2016). Role of insects in pollination of mango trees. International Research Journal of Biological Sciences 5(1), 1–8.

[brv70017-bib-0250] * Kurahashi, H. (1991). The calyptrate muscoid flies collected on weather ships located at the ocean weather stations. Japanese Journal of Sanitary Zoology 42(1), 53–55.

[brv70017-bib-0251] Kurahashi, H. (1997). Witnessing hundreds of *Calliphora nigribarbis* in migratory flight and landing in Nagasaki, Western Japan. Medical Entomology and Zoology 48(1), 55–58. 10.7601/mez.48.55_1.

[brv70017-bib-0252] * Kurahashi, H. , Kawai, S. & Shudo, C. (1991). Seasonal migration of Japanese blow flies, *Aldrichina grahami* (Aldrich) and *Calliphora nigribarbis* Vollenhoven, observed by a mark and recapture method on Hachijo Island, Tokyo. Japanese Journal of Sanitary Zoology 42(1), 57–59.

[brv70017-bib-0253] Lack, D. & Lack, E. (1951). Migration of insects and birds through a Pyrenean pass. The Journal of Animal Ecology 20(1), 63–67. 10.2307/1644.

[brv70017-bib-0254] * Lather, M. , Mallick, P. K. , Sharma, D. , Kale, S. , Dang, A. S. , Adak, T. & Singh, O. P. (2022). Population genetic structure of the malaria vector *Anopheles fluviatilis* species T (Diptera: Culicidae) in India. Medical and Veterinary Entomology 36(2), 194–202. 35182085 10.1111/mve.12566

[brv70017-bib-0255] * Laurence, B. R. (1955). The ecology of some British Sphaeroceridae (Borboridae, Diptera). The Journal of Animal Ecology 24, 187–199.

[brv70017-bib-0256] Lebl, K. , Zittra, C. , Silbermayr, K. , Obwaller, A. , Berer, D. , Brugger, K. , Walter, M. , Pinior, B. , Fuehrer, H.‐P. & Rubel, F. (2015). Mosquitoes (Diptera: Culicidae) and their relevance as disease vectors in the city of Vienna, Austria. Parasitology Research 114, 707–713. 25468380 10.1007/s00436-014-4237-6 PMC4303709

[brv70017-bib-0257] * Lefebvre, V. , Fontaine, C. , Villemant, C. & Daugeron, C. (2014). Are empidine dance flies major flower visitors in alpine environments? A case study in the Alps, France. Biology Letters 10(11), 20140742. 10.1098/rsbl.2014.0742. 25376804 PMC4261866

[brv70017-bib-0258] * Lemic, D. , Benítez, H. A. , Bjeliš, M. , Órdenes‐Claveria, R. , Ninčević, P. , Mikac, K. M. & Živković, I. P. (2020). Agroecological effect and sexual shape dimorphism in medfly *Ceratitis capitata* (Diptera: Tephritidae) an example in Croatian populations. Zoologischer Anzeiger 288, 118–124.

[brv70017-bib-0259] * Levine, R. S. , Peterson, A. T. & Benedict, M. Q. (2004). Distribution of members of *Anopheles quadrimaculatus* Say sl (Diptera: Culicidae) and implications for their roles in malaria transmission in the United States. Journal of Medical Entomology 41(4), 607–613. 10.1603/0022-2585-41.4.607. 15311451

[brv70017-bib-0260] Li, X. , Wu, M. , Ma, J. , Gao, B. , Wu, Q. , Chen, A. , Liu, J. , Jiang, Y. , Zhai, B. & Early, R. (2020). Prediction of migratory routes of the invasive fall armyworm in eastern China using a trajectory analytical approach. Pest Management Science 76(2), 454–463. 10.1002/ps.5530. 31237729

[brv70017-bib-0261] Liu, M. , Wang, X. , Ma, L. , Cao, L. , Liu, H. , Pu, D. & Wei, S. (2019). Genome‐wide developed microsatellites reveal a weak population differentiation in the hoverfly *Eupeodes corollae* (Diptera: Syrphidae) across China. PLoS One 14(9), e0215888. 31557189 10.1371/journal.pone.0215888 PMC6762071

[brv70017-bib-0262] Losey, J. E. & Vaughan, M. (2006). The economic value of ecological services provided by insects. Bioscience 56(4), 311–323. 10.1641/0006-3568(2006)56[311:tevoes]2.0.co;2.

[brv70017-bib-0263] Luder, K. , Knop, E. & Menz, M. H. M. (2018). Contrasting responses in community structure and phenology of migratory and non‐migratory pollinators to urbanization. Diversity and Distributions 24(7), 919–927.

[brv70017-bib-0264] Luo, L. , Xia, H. & Lu, B.‐R. (2019). Crop breeding for drought resistance. Frontiers in Plant Science 10(314), 1–2. 30949189 10.3389/fpls.2019.00314 PMC6435568

[brv70017-bib-0265] Luschi, P. , Benhamou, S. , Girard, C. , Ciccione, S. , Roos, D. , Sudre, J. & Benvenuti, S. (2007). Marine turtles use geomagnetic cues during open‐sea homing. Current Biology 17(2), 126–133. 17240337 10.1016/j.cub.2006.11.062

[brv70017-bib-0266] Lysenkov, S. N. (2009). On the estimation of the influence of the character of insect pollinators movements on the pollen transfer dynamics. Entomological Review 89(2), 143–149.

[brv70017-bib-0267] * MacFarlane, J. R. , East, R. W. , Drew, R. A. I. & Betlinski, G. A. (1987). Dispersal of irradiated Queensland fruit‐flies, Dacus‐Tryoni (Froggatt)(Diptera, Tephritidae), in Southeastern Australia. Australian Journal of Zoology 35(3), 275–281. 10.1071/ZO9870275.

[brv70017-bib-0268] * Magor, J. I. & Rosenberg, L. J. (1980). Studies of winds and weather during migrations of *Simulium damnosum* Theobald (Diptera: Simuliidae), the vector of onchocerciasis in West Africa. Bulletin of Entomological Research 70(4), 693–716. 10.1017/S0007485300007987.

[brv70017-bib-0269] * Malijan, R. P. B. , Mechan, F. , Braganza Jr, J. C. , Valle, K. M. R. , Salazar, F. V. , Torno, M. M. , Aure, W. E. , Bacay, B. A. , Espino, F. E. , Torr, S. J. & Fornace, K. M. (2021). The seasonal dynamics and biting behavior of potential *anopheles* vectors of *Plasmodium knowlesi* in Palawan, Philippines. Parasites & Vectors 14(1), 357. 34233742 10.1186/s13071-021-04853-9 PMC8261946

[brv70017-bib-0270] * Manin, B. O. , Drakeley, C. J. & Chua, T. H. (2018). Mitochondrial variation in subpopulations of *Anopheles balabacensis* Baisas in Sabah, Malaysia (Diptera: Culicidae). PLoS One 13(8), e0202905. 30138386 10.1371/journal.pone.0202905 PMC6107281

[brv70017-bib-0271] * Marchiori, C. H. (2022 *a*). Contribution to the knowledge of the family Bibionidae (Insecta: Diptera). International Journal of Science and Research Archive 6(2), 52–80.

[brv70017-bib-0272] * Marchiori, C. H. (2022 *b*). Family Sepsidae associated with the decomposition of organic matter (Insecta: Diptera). Open Access Research Journal of Science and Technology 5(2), 16–38.

[brv70017-bib-0273] * Marchiori, C. H. (2022 *c*). Importance of Sphaeroceridae family as accelerators of the putrefaction process, nutrient recycling and in its association with the cadaveric decomposition process (Insecta: Diptera). Open Access Research Journal of Science and Technology 4(2), 28–42.

[brv70017-bib-0274] * Marchiori, C. H. (2023 *a*). Study of the biological and taxonomic characteristics of the families Megamerinidae, Nemestrinidae and Therevidae (Insecta: Diptera). Open Access Research Journal of Biology and Pharmacy 7(2), 43–74.

[brv70017-bib-0275] * Marchiori, C. H. (2023 *b*). The characteristics of the families Anisopodidae and Mycetophilidae (Insecta: Diptera). International Journal of Life Science Research Archive 5(2), 96–108.

[brv70017-bib-0276] Marshall, S. A. (2012). FLIES: The Natural History and Diversity of Diptera, p. 616. Firefly Press Ltd., Ontario, Canada.

[brv70017-bib-0277] * Martens, C. (2020). Use of the Helgoland Bird Trap in Kabli (Estonia) to investigate seasonal migration of Diptera (Syrphidae, Muscidae, Calliphoridae and Polleniidae). Bulletin de la Société royale belge d'Entomologie 156, 247–253.

[brv70017-bib-0278] * Martens, C. , Dekonink, W. & Grootaert, P. (2011). Muscidae and Syrphidae (Diptera) collected by window‐trapping at the IJzer estuary (Belgian coast). Bulletin van de Koninklijke Belgische Vereniging voor Entomologie 147, 225–232.

[brv70017-bib-0279] * Martínez‐Calabuig, N. , Panadero, R. , Remesar, S. , García‐Dios, D. , Saldaña, A. , Díaz, P. , Prieto, A. , Díez‐Baños, P. , Morrondo, P. & López, C. M. (2023). Pedicle myiasis by Lucilia caesar (Diptera, Calliphoridae): an emerging disease in roe deer from north‐western Spain. Medical and Veterinary Entomology 37(3), 581–585. 10.1111/mve.12654. 37042792

[brv70017-bib-0280] Massy, R. , Hawkes, W. , Weston, S. , Doyle, T. & Wotton, K. R. (2024). Enhanced flight performance in hoverfly migrants. iScience 27(12), 1–9. 10.1016/j.isci.2024.111345 PMC11617951 39640581

[brv70017-bib-0281] Massy, R. , Hawkes, W. L. S. , Doyle, T. , Troscianko, J. , Menz, M. H. M. , Roberts, N. W. , Chapman, J. W. & Wotton, K. R. (2021). Hoverflies use a time‐compensated sun compass to orientate during autumn migration. Proceedings of the Royal Society B 288(1959), 20211805. 34547904 10.1098/rspb.2021.1805 PMC8456149

[brv70017-bib-0282] * Mayoke, A. , Ouma, J. O. , Mireji, P. O. , Omondi, S. F. , Muya, S. M. , Itoua, A. , Okoth, S. O. & Bateta, R. (2021). Population structure and migration patterns of the tsetse Fly *Glossina fuscipes* in Congo‐Brazzaville. The American Journal of Tropical Medicine and Hygiene 104(3), 917. 10.4269/ajtmh.20-0774 PMC7941806 33372648

[brv70017-bib-0283] Mayor, S. J. , Guralnick, R. P. , Tingley, M. W. , Otegui, J. , Withey, J. C. , Elmendorf, S. C. , Andrew, M. E. , Leyk, S. , Pearse, I. S. & Schneider, D. C. (2017). Increasing phenological asynchrony between spring green‐up and arrival of migratory birds. Scientific Reports 7(1), 1902. 28507323 10.1038/s41598-017-02045-z PMC5432526

[brv70017-bib-0284] * Mcdonald, P. T. , Hilburn, L. R. & Kunz, S. E. (1987). Genetic similarities among natural populations of the horn fly (Diptera: Muscidae). Annals of the Entomological Society of America 80(2), 288–292.

[brv70017-bib-0285] * McEvey, S. F. (1981). Drosophilidae (Insecta: Diptera) of three Torres Strait Islands, with description of a new species of drosophila. Australian Journal of Zoology 29(6), 907–919.

[brv70017-bib-0286] * McGregor, B. L. , Stenn, T. , Sayler, K. A. , Blosser, E. M. , Blackburn, J. K. , Wisely, S. M. & Burkett‐Cadena, N. D. (2019). Host use patterns of *Culicoides* spp. biting midges at a big game preserve in Florida, USA, and implications for the transmission of orbiviruses. Medical and Veterinary Entomology 33(1), 110–120. 10.1111/mve.12331. 30063255

[brv70017-bib-0287] * Meier, R. , Kotrba, M. & Barber, K. N. (1997). A comparative study of the egg, first‐instar larva, puparium, female reproductive system, and natural history of Curtonotum helvum (Curtonotidae, Ephydroidea, Diptera). American Museum Novitates 3219, 1–20.

[brv70017-bib-0288] Menz, M. H. M. , Brown, B. v. & Wotton, K. R. (2019a). Quantification of migrant hoverfly movements (Diptera: Syrphidae) on the west coast of North America. Royal Society Open Science 6(4), 190153. 31183151 10.1098/rsos.190153 PMC6502382

[brv70017-bib-0289] Menz, M. H. M. , Reynolds, D. R. , Gao, B. , Hu, G. , Chapman, J. W. & Wotton, K. R. (2019 *b*). Mechanisms and consequences of partial migration in insects. Frontiers in Ecology and Evolution 7(403), 403. 10.3389/fevo.2019.00403.

[brv70017-bib-0290] Menz, M. H. M. , Scacco, M. , Bürki‐Spycher, H.‐M. , Williams, H. J. , Reynolds, D. R. , Chapman, J. W. & Wikelski, M. (2022). Individual tracking reveals long‐distance flight‐path control in a nocturnally migrating moth. Science 377(6607), 764–768. 35951704 10.1126/science.abn1663

[brv70017-bib-0291] * Mesmin, X. , Vincent, M. , Tricault, Y. , Estorgues, V. , Daniel, L. , Cortesero, A. M. , Faloya, V. & Le Ralec, A. (2019). Assessing the relationship between pest density and plant damage: a case study with the belowground herbivore *Delia radicum* (Diptera: Anthomyiidae) on broccoli. Applied Entomology and Zoology 54, 155–165.

[brv70017-bib-0292] Meyer, B. , Jauker, F. & Steffan‐Dewenter, I. (2009). Contrasting resource‐dependent responses of hoverfly richness and density to landscape structure. Basic and Applied Ecology 10(2), 178–186.

[brv70017-bib-0293] Meyer, W. B. & Turner, B. L. (1992). Human population growth and global land‐use/cover change. Annual Review of Ecology and Systematics 23(1), 39–61. 10.1146/annurev.es.23.110192.000351.

[brv70017-bib-0294] * Miao, J. , Wu, Y. Q. , Gong, Z. J. , He, Y. Z. , Duan, Y. & Jiang, Y. L. (2013). Long‐distance wind‐borne dispersal of *Sitodiplosis mosellana* Géhin (Diptera: Cecidomyiidae) in Northern China. Journal of Insect Behavior 26(1), 120–129. 10.1007/s10905-012-9346-4.

[brv70017-bib-0295] Mignotte, A. , Garros, C. , Dellicour, S. , Jacquot, M. , Gilbert, M. , Gardès, L. , Balenghien, T. , Duhayon, M. , Rakotoarivony, I. , de Wavrechin, M. & Huber, K. (2021). High dispersal capacity of *Culicoides obsoletus* (Diptera: Ceratopogonidae), vector of bluetongue and Schmallenberg viruses, revealed by landscape genetic analyses. Parasites & Vectors 14(1), 1–14. 33536057 10.1186/s13071-020-04522-3 PMC7860033

[brv70017-bib-0296] Mihok, S. & Clausen, P. H. (1996). Feeding habits of *Stomoxys* spp. stable flies in a Kenyan forest. Medical and Veterinary Entomology 10(4), 392–394. 8994143 10.1111/j.1365-2915.1996.tb00762.x

[brv70017-bib-0297] Miličić, M. , Vujić, A. & Cardoso, P. (2018). Effects of climate change on the distribution of hoverfly species (Diptera: Syrphidae) in Southeast Europe. Biodiversity and Conservation 27(5), 1173–1187.

[brv70017-bib-0298] * Miller, R. M. (1977). Ecology of Lauxaniidae (Diptera: Acalyptratae) 1. Old and new rearing records with biological notes and discussion. Annals of the Natal Museum 23(1), 215–238.

[brv70017-bib-0299] * Mitsui, H. , Beppu, K. & Kimura, M. T. (2010). Seasonal life cycles and resource uses of flower‐and fruit‐feeding drosophilid flies (Diptera: Drosophilidae) in central Japan. Entomological Science 13(1), 60–67. 10.1111/j.1479-8298.2010.00372.x.

[brv70017-bib-0300] * Mitsui, H. & Nakayama, H. (2012). Seasonal occurrence of phorid flies (Diptera) attracted to rotten flesh in and near Tokyo. Medical Entomology and Zoology 63(3), 205–208.

[brv70017-bib-0301] Moerkens, R. , Boonen, S. , Wäckers, F. L. & Pekas, A. (2021). Aphidophagous hoverflies reduce foxglove aphid infestations and improve seed set and fruit yield in sweet pepper. Pest Management Science 77(6), 2690–2696. 33638225 10.1002/ps.6342

[brv70017-bib-0302] * Moffat, A. (2023). Tipula spp. pest status in Irish and Scottish agriculture: new insights into a long‐established pest. *Doctoral Dissertation*.

[brv70017-bib-0303] * Mohr, C. O. (1943). Cattle droppings as ecological units. Ecological Monographs 13(3), 275–298.

[brv70017-bib-0304] Mramba, F. , Broce, A. B. & Zurek, L. (2007). Vector competence of stable flies, *Stomoxys calcitrans* L.(Diptera: Muscidae), for *Enterobacter sakazakii* . Journal of Vector Ecology 32(1), 134–139. 10.3376/1081-1710(2007)32[134:vcosfs]2.0.co;2. 17633434

[brv70017-bib-0305] * Mutamiswa, R. , Nyamukondiwa, C. , Chikowore, G. & Chidawanyika, F. (2021). Overview of oriental fruit fly, *Bactrocera dorsalis* (Hendel) (Diptera: Tephritidae) in Africa: from invasion, bio‐ecology to sustainable management. Crop Protection 141, 105492. 10.1016/j.cropro.2020.105492.

[brv70017-bib-0306] * Muturi, E. J. , Muriu, S. , Shililu, J. , Mwangangi, J. M. , Jacob, B. G. , Mbogo, C. , Githure, J. & Novak, R. J. (2008). Blood‐feeding patterns of *Culex quinquefasciatus* and other culicines and implications for disease transmission in Mwea rice scheme, Kenya. Parasitology Research 102, 1329–1335. 18297310 10.1007/s00436-008-0914-7

[brv70017-bib-0307] Nabeshima, T. , Loan, H. T. K. , Inoue, S. , Sumiyoshi, M. , Haruta, Y. , Nga, P. T. , Huoung, V. T. Q. , del Carmen Parquet, M. , Hasebe, F. & Morita, K. (2009). Evidence of frequent introductions of Japanese encephalitis virus from south‐east Asia and continental east Asia to Japan. Journal of General Virology 90(4), 827–832. 19264633 10.1099/vir.0.007617-0

[brv70017-bib-0308] * Nartshuk, E. P. (2014). Grass‐fly larvae (Diptera, Chloropidae): diversity, habitats, and feeding specializations. Entomological Review 94, 514–525.

[brv70017-bib-0309] * Nchoutpouen, E. , Talipouo, A. , Djiappi‐Tchamen, B. , Djamouko‐Djonkam, L. , Kopya, E. , Ngadjeu, C. S. , Doumbe‐Belisse, P. , Awono‐Ambene, P. , Kekeunou, S. , Wondji, C. S. & Antonio‐Nkondjio, C. (2019). *Culex* species diversity, susceptibility to insecticides and role as potential vector of *Lymphatic filariasis* in the city of Yaoundé, Cameroon. PLoS Neglected Tropical Diseases 13(4), e0007229. 10.1371/journal.pntd.0007229. 30943198 PMC6464241

[brv70017-bib-0310] * Nielsen, L. B. & Nielsen, B. O. (2002). Density and phenology of soil gallmidges (Diptera: Cecidomyiidae) in arable land. Pedobiologia 46(1), 1–14. 10.1078/0031-4056-00108.

[brv70017-bib-0311] * Nielsen, T. R. (2009). A migration of *Eristalis similis* (Fallén, 1817)(Diptera, Syrphidae) at Lindesnes, South Norway in 2009. Norwegian Journal Of Entomology 56, 74.

[brv70017-bib-0312] Nielsen, T. R. , Andreassen, A. T. & Leendertse, A. S. S. (2010). A migration of the Hoverfly *Helophilus trivittatus* (Fabricius, 1805) (Diptera, Syrphidae) to SW Norway in 2010. Norwegian Journal Of Entomology 57, 136–138.

[brv70017-bib-0313] Nilssen, A. C. & Anderson, J. R. (1995). Flight capacity of the reindeer warble fly, *Hypoderma tarandi* (L.), and the reindeer nose bot fly, *Cephenemyia trompe* (Modeer) (Diptera: Oestridae). Canadian Journal of Zoology 73(7), 1228–1238.

[brv70017-bib-0314] * Nishida, T. (1963). Zoogeographical and ecological studies of Dacus cucurbitae (Diptera‐Tephritidae) in India. Technical Bulletin 54 1–28.

[brv70017-bib-0315] * Noma, T. & Brewer, M. J. (2008). Seasonal abundance of resident parasitoids and predatory flies and corresponding soybean aphid densities, with comments on classical biological control of soybean aphid in the Midwest. Journal of Economic Entomology 101(2), 278–287. 18459389 10.1603/0022-0493(2008)101[278:saorpa]2.0.co;2

[brv70017-bib-0316] * North, A. R. & Godfray, H. C. J. (2018). Modelling the persistence of mosquito vectors of malaria in Burkina Faso. Malaria Journal 17, 1–15. 29609598 10.1186/s12936-018-2288-3 PMC5879775

[brv70017-bib-0317] Odermatt, J. , Frommen, J. G. & Menz, M. H. M. (2017). Consistent behavioural differences between migratory and resident hoverflies. Animal Behaviour 127, 187–195.

[brv70017-bib-0318] * Onder, Z. , Yildirim, A. , Duzlu, O. , Arslan, M. O. , Sari, B. , Tasci, G. T. , Ciloglu, A. , Aydin, N. P. , Inci, A. & Adler, P. H. (2019). Molecular characterization of black flies (Diptera: Simuliidae) in areas with pest outbreaks and simuliotoxicosis in Northeast Anatolia region, Turkey. Acta Tropica 199, 105149. 31422094 10.1016/j.actatropica.2019.105149

[brv70017-bib-0319] * Ordax, M. , Piquer‐Salcedo, J. E. , Santander, R. D. , Sabater‐Muñoz, B. , Biosca, E. G. , López, M. M. & Marco‐Noales, E. (2015). Medfly *Ceratitis capitata* as potential vector for fire blight pathogen Erwinia amylovora: survival and transmission. PLoS One 10(5), e0127560. 25978369 10.1371/journal.pone.0127560 PMC4433354

[brv70017-bib-0320] * Orford, K. A. , Vaughan, I. P. & Memmott, J. (2015). The forgotten flies: the importance of non‐syrphid Diptera as pollinators. Proceedings of the Royal Society B: Biological Sciences 282(1805), 20142934. 10.1098/rspb.2014.2934 PMC4389612 25808886

[brv70017-bib-0321] * Ouin, A. , Menozzi, P. , Coulon, M. , Hamilton, A. J. , Sarthou, J. P. , Tsafack, N. , Vialatte, A. & Ponsard, S. (2011). Can deuterium stable isotope values be used to assign the geographic origin of an auxiliary hoverfly in south‐western France? Rapid Communications in Mass Spectrometry 25(19), 2793–2798. 21913257 10.1002/rcm.5127

[brv70017-bib-0322] * Parchami‐Araghi, M. & Gilasian, Ù. E. (2020). Review of Muscina Robineau‐Desvoidy (Diptera: Muscidae) in Iran. Journal of Entomological Society of Iran 40(1), 95–103.

[brv70017-bib-0323] Parker, D. J. , Burton, R. R. , Diongue‐Niang, A. , Ellis, R. J. , Felton, M. , Taylor, C. M. , Thorncroft, C. D. , Bessemoulin, P. & Tompkins, A. M. (2005). The diurnal cycle of the West African monsoon circulation. Quarterly Journal of the Royal Meteorological Society 131(611), 2839–2860.

[brv70017-bib-0324] * Parry, H. R. , Eagles, D. & Kriticos, D. J. (2015). Simulation modelling of long‐distance windborne dispersal for invasion ecology. In Pest Risk Modelling and Mapping for Invasive Alien Species, pp. 49–64. CABI, Wallingford.

[brv70017-bib-0325] Pasanen, M. (2020). Characterization of Pectobacterium strains causing soft rot and blackleg of potato in Finland. Doctoral Thesis: Faculty of Agriculture and Forestry of the University of Helsinki.

[brv70017-bib-0326] * Pasek, J. E. (1988). Influence of wind and windbreaks on local dispersal of insects. Insects. Agriculture, Ecosystems & Environment 22, 539–554.

[brv70017-bib-0327] * Peck, S. B. (1994). Aerial dispersal of insects between and to islands in the Galapagos archipelago, Ecuador. Annals of the Entomological Society of America 87(2), 218–224.

[brv70017-bib-0328] * Pedersen, E. T. (1982). Flere Syrphidae (Diptera) fra Anholt. Flora og Fauna 88, 15–17.

[brv70017-bib-0329] Pedersen, L. , Thorup, K. & Tøttrup, A. P. (2019). Annual GPS tracking reveals unexpected wintering area in a long‐distance migratory songbird. Journal of Ornithology 160(1), 265–270.

[brv70017-bib-0330] * Pedro, P. M. & Sallum, M. A. (2009). Spatial expansion and population structure of the neotropical malaria vector, *Anopheles darlingi* (Diptera: Culicidae). Biological Journal of the Linnean Society 97(4), 854–866.

[brv70017-bib-0331] Pérez‐Bañón, C. , Juan, A. , Petanidou, T. , Marcos‐García, M. A. & Crespo, M. B. (2003). The reproductive ecology of *Medicago citrina* (Font Quer) Greuter (Leguminosae): a bee‐pollinated plant in Mediterranean islands where bees are absent. Plant Systematics and Evolution 241(1), 29–46. 10.1007/s00606-003-0004-3.

[brv70017-bib-0332] Pérez‐Bañón, C. , Petanidou, T. & Marcos‐García, M. (2007). Pollination in small islands by occasional visitors: the case of *Daucus carota* subsp. *commutatus* (Apiaceae) in the Columbretes archipelago, Spain. Plant Ecology 192(1), 133–151.

[brv70017-bib-0333] Pineda, A. & Marcos‐García, M. (2008). Evaluation of several strategies to increase the residence time of *Episyrphus balteatus* (Diptera, Syrphidae) releases in sweet pepper greenhouses. Annals of Applied Biology 152(3), 271–276. 10.1111/j.1744-7348.2008.00215.x.

[brv70017-bib-0334] * Psarev, A. M. (2001). Note about a diurnal rhythmicity of coprophilous flies and their parasitoids on mountain pastures. International Journal of Dipterological Research 12(1), 53–56.

[brv70017-bib-0335] * Purse, B. V. , Falconer, D. , Sullivan, M. J. , Carpenter, S. , Mellor, P. S. , Piertney, S. B. , Mordue, A. J. , Albon, S. , Gunn, G. J. & Blackwell, A. (2012). Impacts of climate, host and landscape factors on Culicoides species in Scotland. Medical and Veterinary Entomology 26(2), 168–177. 10.1111/j.1365-2915.2011.00991.x. 22103842

[brv70017-bib-0336] * Qin, Y. , Krosch, M. N. , Schutze, M. K. , Zhang, Y. , Wang, X. , Prabhakar, C. S. , Susanto, A. , Hee, A. K. W. , Ekesi, S. & Badji, K. (2018). Population structure of a global agricultural invasive pest, *Bactrocera dorsalis* (Diptera: Tephritidae). Evolutionary Applications 11(10), 1990–2003. 10.1111/eva.12701. 30459843 PMC6231469

[brv70017-bib-0337] Rader, R. , Cunningham, S. A. , Howlett, B. G. & Inouye, D. W. (2020). Non‐bee insects as visitors and pollinators of crops: biology, ecology, and management. Annual Review of Entomology 65, 391–407. 10.1146/annurev-ento-011019-025055 31610136

[brv70017-bib-0338] Rader, R. , Edwards, W. , Westcott, D. A. , Cunningham, S. A. & Howlett, B. G. (2011). Pollen transport differs among bees and flies in a human‐modified landscape. Diversity and Distributions 17(3), 519–529.

[brv70017-bib-0339] Raftery, A. E. , Zimmer, A. , Frierson, D. M. W. , Startz, R. & Liu, P. (2017). Less than 2 C warming by 2100 unlikely. Nature Climate Change 7(9), 637–641. 10.1038/nclimate3352 PMC6070153 30079118

[brv70017-bib-0340] * Ratovonjato, J. , Olive, M. M. , Tantely, L. M. , Andrianaivolambo, L. , Tata, E. , Razainirina, J. , Jeanmaire, E. , Reynes, J. M. & Elissa, N. (2011). Detection, isolation, and genetic characterization of Rift Valley fever virus from *Anopheles* (Anopheles) *coustani*, *Anopheles* (Anopheles) *squamosus*, and *Culex* (Culex) *antennatus* of the Haute Matsiatra region, Madagascar. Vector‐Borne and Zoonotic Diseases 11(6), 753–759. 10.1089/vbz.2010.0031. 21028960

[brv70017-bib-0341] * Raymond, L. , Plantegenest, M. , Gauffre, B. , Sarthou, J.‐P. & Vialatte, A. (2013a). Lack of genetic differentiation between contrasted overwintering strategies of a major pest predator *Episyrphus balteatus* (Diptera: Syrphidae): implications for biocontrol. PLoS One 8(9), e72997. 10.1371/journal.pone.0072997. 24023799 PMC3759392

[brv70017-bib-0342] * Raymond, L. , Plantegenest, M. & Vialatte, A. (2013 *b*). Migration and dispersal may drive to high genetic variation and significant genetic mixing: the case of two agriculturally important, continental hoverflies (*Episyrphus balteatus* and *Sphaerophoria scripta*). Molecular Ecology 22(21), 5329–5339. 24138027 10.1111/mec.12483

[brv70017-bib-0343] Raymond, L. , Sarthou, J.‐P. , Plantegenest, M. , Gauffre, B. , Ladet, S. & Vialatte, A. (2014). Immature hoverflies overwinter in cultivated fields and may significantly control aphid populations in autumn. Agriculture, Ecosystems & Environment 185, 99–105. 10.1016/j.agee.2013.12.019.

[brv70017-bib-0344] Raymond, M. & Pasteur, N. (1996). Evolution of insecticide resistance in the mosquito *Culex pipiens*: the migration hypothesis of amplified esterase genes. In Molecular Genetics and Evolution of Pesticide Resistance (Volume 645), pp. 90–96. American Chemical Society, Washington, DC.

[brv70017-bib-0345] * Recinos‐Aguilar, Y. M. , García‐García, M. D. , Malo, E. A. , Cruz‐López, L. & Rojas, J. C. (2019). The colonization of necrophagous larvae accelerates the decomposition of chicken carcass and the emission of volatile attractants for blowflies (Diptera: Calliphoridae). Journal of Medical Entomology 56(6), 1590–1597. 31265073 10.1093/jme/tjz104

[brv70017-bib-0346] * Reeves, L. E. , Holderman, C. J. , Blosser, E. M. , Gillett‐Kaufman, J. L. , Kawahara, A. Y. , Kaufman, P. E. & Burkett‐Cadena, N. D. (2018). Identification of *Uranotaenia sapphirina* as a specialist of annelids broadens known mosquito host use patterns. Communications Biology 1(1), 92. 10.1038/s42003-018-0096-5. 30271973 PMC6123777

[brv70017-bib-0347] * Reissig, W. H. , Weires, R. W. , Forshey, C. G. , Roelofs, W. L. , Lamb, R. C. , Aldwinckle, H. S. & Alm, S. R. (1984). Management of the apple maggot, *Rhagoletis pomonella* (Walsh)(Diptera: Tephritidae), in disease‐resistant dwarf and semi‐dwarf apple trees. Environmental Entomology 13(3), 684–690. 10.1093/ee/13.3.684.

[brv70017-bib-0348] * Ren, Z. X. , Li, D. Z. , Bernhardt, P. & Wang, H. (2011). Flowers of *Cypripedium fargesii* (Orchidaceae) fool flat‐footed flies (Platypezidae) by faking fungus‐infected foliage. Proceedings of the National Academy of Sciences 108(18), 7478–7480. 10.1073/pnas.1103384108. PMC3088628 21502502

[brv70017-bib-0349] Reppert, S. M. (2007). The ancestral circadian clock of monarch butterflies: role in time‐compensated sun compass orientation. Cold Spring Harbor Symposia on Quantitative Biology 72, 113–118. 18419268 10.1101/sqb.2007.72.056

[brv70017-bib-0350] Reppert, S. M. & de Roode, J. C. (2018). Demystifying monarch butterfly migration. Current Biology 28(17), 1009–1022. 10.1016/j.cub.2018.02.067 30205052

[brv70017-bib-0351] Reynolds, S. K. , Clem, C. S. , Fitz‐Gerald, B. & Young, A. D. (2024). A comprehensive review of long‐distance hover fly migration (Diptera: Syrphidae). Ecological Entomology 49(6), 749–767.

[brv70017-bib-0352] Riad, M. H. , Scoglio, C. M. , McVey, D. S. & Cohnstaedt, L. W. (2017). An individual‐level network model for a hypothetical outbreak of Japanese encephalitis in the USA. Stochastic Environmental Research and Risk Assessment 31(2), 353–367.

[brv70017-bib-0353] Ritchie, S. A. & Rochester, W. (2001). Wind‐blown mosquitoes and introduction of Japanese encephalitis into Australia. Emerging Infectious Diseases 7(5), 900–908. 11747709 10.3201/eid0705.017524 PMC2631883

[brv70017-bib-0354] * Rogoff, W. M. , Carbrey, E. G. , Bram, R. A. , Clark, T. B. & Gretz, G. H. (1975). Transmission of Newcastle disease virus by insects: detection in wild *Fannia* spp.(Diptera: Muscidae). Journal of Medical Entomology 12(2), 225–227. 10.1093/jmedent/12.2.225. 1159747

[brv70017-bib-0355] * Roiz, D. , Wilson, A. L. , Scott, T. W. , Fonseca, D. M. , Jourdain, F. , Müller, P. , Velayudhan, R. & Corbel, V. (2018). Integrated *Aedes* management for the control of Aedes‐borne diseases. PLoS Neglected Tropical Diseases 12(12), e0006845. 30521524 10.1371/journal.pntd.0006845 PMC6283470

[brv70017-bib-0356] Rojo, S. , FS, G. , Marcos‐García, M. , Nieto, J. M. & Mier Durante, M. P. (2003). A World Review of Predatory Hoverflies (Diptera, Syrphidae: Syrphinae) and their Prey. CIBIO Ediciones, Alicante, Spain.

[brv70017-bib-0357] * Rojo, S. , Isidro, P. M. , Perez‐Bañón, M. C. & Marcos‐García, M. A. (1997). Revision of the hoverflies (Diptera: Syrphidae) from the Azores archipelago with notes on Macaronesian syrphid fauna. ARQUIPÉLAGO. Ciências Biológicas e Marinhas= Life and Marine Sciences 15, 65–82.

[brv70017-bib-0358] Roth, S. K. & Lindroth, R. L. (1994). Effects of CO_2_‐mediated changes in paper birch and white pine chemistry on gypsy moth performance. Oecologia 98, 133–138. 28313969 10.1007/BF00341464

[brv70017-bib-0359] Roth, S. K. & Lindroth, R. L. (1995). Elevated atmospheric CO_2_: effects on phytochemistry, insect performance and insect‐parasitoid interactions. Global Change Biology 1(3), 173–182.

[brv70017-bib-0360] * Rotheray, G. E. & Rotheray, G. E. (2019). Saprophagy, developing on decay. In Ecomorphology of Cyclorrhaphan Larvae (Diptera), pp. 141–173. Springer International Publishing, Cham.

[brv70017-bib-0361] Runge, C. A. , Martin, T. G. , Possingham, H. P. , Willis, S. G. & Fuller, R. A. (2014). Conserving mobile species. Frontiers in Ecology and the Environment 12(7), 395–402.

[brv70017-bib-0362] * Rupp, T. , Oelschlägel, B. , Rabitsch, K. , Mahfoud, H. , Wenke, T. , Disney, R. H. L. , Neinhuis, C. , Wanke, S. & Dötterl, S. (2021). Flowers of deceptive *Aristolochia microstoma* are pollinated by phorid flies and emit volatiles known from invertebrate carrion. Frontiers in Ecology and Evolution 9, 658441. 10.3389/fevo.2021.658441.

[brv70017-bib-0363] * Russo, C. A. , Mello, B. , Frazão, A. & Voloch, C. M. (2013). Phylogenetic analysis and a time tree for a large drosophilid data set (Diptera: Drosophilidae). Zoological Journal of the Linnean Society 169(4), 765–775. 10.1111/zoj.12062.

[brv70017-bib-0364] * Rygg, T. D. (1966). Flight of *Oscinella frit* L. (Diptera, Chloropidae) females in relation to age and ovary development. Entomologia Experimentalis et Applicata 9(1), 74–84.

[brv70017-bib-0365] * Sabrosky, C. W. (1936). A review of the Nearctic species of *Chloropisca* (Diptera, Chloropidae). The Canadian Entomologist 68(8), 170–177.

[brv70017-bib-0366] * Sabrosky, C. W. (1956). Additions to the knowledge of Old World Asteiidae. Revue francaise d'entomologie (Nouvelle Serie) 23, 216–243.

[brv70017-bib-0367] * Sahu, S. S. , Gunasekaran, K. , Krishnamoorthy, N. , Vanamail, P. , Mathivanan, A. , Manonmani, A. & Jambulingam, P. (2017). Bionomics of *Anopheles fluviatilis* and *Anopheles culicifacies* (Diptera: Culicidae) in relation to malaria transmission in East‐Central India. Journal of Medical Entomology 54(4), 821–830. 28399290 10.1093/jme/tjx065 PMC5850663

[brv70017-bib-0368] Sainz‐Borgo, C. , Miranda, J. & Lentino, M. (2020). Composition of bird community in Portachuelo Pass (Henri Pittier National Park, Venezuela). Journal of Caribbean Ornithology 33, 1–14.

[brv70017-bib-0369] * Saito, T. (2004). Insecticide susceptibility of the leafminer, *Chromatomyia horticola* (Goureau)(Diptera: Agromyzidae). Applied Entomology and Zoology 39(2), 203–208. 10.1303/aez.2004.203.

[brv70017-bib-0370] Samplonius, J. M. , Atkinson, A. , Hassall, C. , Keogan, K. , Thackeray, S. J. , Assmann, J. J. , Burgess, M. D. , Johansson, J. , Macphie, K. H. & Pearce‐Higgins, J. W. (2021). Strengthening the evidence base for temperature‐mediated phenological asynchrony and its impacts. Nature Ecology & Evolution 5(2), 155–164. 10.1038/s41559-020-01357-0. 33318690

[brv70017-bib-0371] Sánchez‐Bayo, F. & Wyckhuys, K. A. G. (2019). Worldwide decline of the entomofauna: a review of its drivers. Biological Conservation 232, 8–27.

[brv70017-bib-0372] * Sanders, C. J. , Selby, R. , Carpenter, S. & Reynolds, D. R. (2011). High‐altitude flight of Culicoides biting midges. Bulletin of Entomological Research 94, 123–136. 10.1136/vr.d4245 21778146

[brv70017-bib-0373] * Sanogo, Z. L. , Yaro, A. S. , Dao, A. , Diallo, M. , Yossi, O. , Samaké, D. , Krajacich, B. J. , Faiman, R. & Lehmann, T. (2021). The effects of high‐altitude windborne migration on survival, oviposition, and blood‐feeding of the African malaria mosquito, *Anopheles gambiae* sl (Diptera: Culicidae). Journal of Medical Entomology 58(1), 343–349. 32667040 10.1093/jme/tjaa137 PMC7801746

[brv70017-bib-0374] Satterfield, D. A. , Sillett, T. S. , Chapman, J. W. , Altizer, S. & Marra, P. P. (2020). Seasonal insect migrations: massive, influential, and overlooked. Frontiers in Ecology and the Environment 18(6), 335–344.

[brv70017-bib-0375] * Schmid, U. (1999). Schwebfliegen auf dem Mittelmeer (Diptera, Syrphidae). Volucella 4(1/2), 167–170.

[brv70017-bib-0376] * Sedivy, J. (1994). Seasonal migration of wheat midges (*Contarinia tritici* and *Sitodiplosis mosellana* (Diptera, Cecidomyidae)). Ochrana Rostlin‐UZPI Czech Republic 30(1), 1–9.

[brv70017-bib-0377] * Self, L. S. , Shin, H. K. , Kim, K. H. , Lee, K. W. , Chow, C. Y. & Hong, H. K. (1973). Ecological studies on *Culex tritaeniorhynchus* as a vector of Japanese encephalitis. Bulletin of the World Health Organization 49(1), 41–47. 4363396 PMC2481081

[brv70017-bib-0378] * Sellers, R. F. (1980). Weather, host and vector–their interplay in the spread of insect‐borne animal virus diseases. Epidemiology & Infection 85(1), 65–102. 10.1017/s0022172400027108 PMC2134001 6131919

[brv70017-bib-0379] * Service, M. W. (1980). Effects of wind on the behaviour and distribution of mosquitoes and blackflies. International Journal of Biometeorology 24, 347–353.

[brv70017-bib-0380] * Service, M. W. (1997). Mosquito (Diptera: Culicidae) dispersal—the long and short of it. Journal of Medical Entomology 34(6), 579–588. 9439109 10.1093/jmedent/34.6.579

[brv70017-bib-0381] Shannon, H. J. (1926). A preliminary report on the seasonal migrations of insects. Journal of the New York Entomological Society 34(2), 199–205.

[brv70017-bib-0382] * Shaw, J. G. , Sanchez‐Riviello, M. , Spishakoff, L. M. , Trujillo, G. P. & Loppez, D. F. (1967). Dispersal and migration of tepa‐sterilized Mexican fruit flies. Journal of Economic Entomology 60(4), 992–994.

[brv70017-bib-0383] * Shi, C. H. , Yang, Y. T. , Han, H. L. , Chen, J. X. , Wu, Q. J. , Xu, B. Y. & Zhang, Y. J. (2016). Population dynamics and summer and winter habitats of *Bradysia odoriphaga* in the Beijing area. Chinese Journal of Applied Entomology 53, 1174–1183.

[brv70017-bib-0384] * Shi, W. , Kerdelhue, C. & Ye, H. (2005). Population genetics of the oriental fruit fly, *Bactrocera dorsalis* (Diptera: Tephritidae), in Yunnan (China) based on mitochondrial DNA sequences. Environmental Entomology 34(4), 977–983. 10.1603/0046-225X-34.4.977.

[brv70017-bib-0385] Shpedt, A. A. , Aksenova, Y. V. , Shayakhmetov, M. R. , Zhulanova, V. N. , Rassypnov, V. A. & Butyrin, M. V. (2019). Soil and ecological evaluation of agro‐chernozems of Siberia. International Transaction Journal of Engineering, Management & Applied Sciences & Technologies 10(3), 309–318.

[brv70017-bib-0386] * Silva, D. C. , Machado, L. P. B. & Mateus, R. P. (2015). Migration rate and genetic diversity of two *Drosophila maculifrons* (Duda, 1927) populations from Highland Araucaria Forest Fragments in Southern Brazil. Brazilian Journal of Biology 75, 254–255. 10.1590/1519-6984.09914 25945646

[brv70017-bib-0387] * Skuhrava, M. (2006). Species richness of gall midges (Diptera: Cecidomyiidae) in the main biogeographical regions of the world. Acta Societatis Zoologicae Bohemicae 69, 327–372.

[brv70017-bib-0388] * Smit, J. T. , Aguiar, A. F. & Wakeham‐Dawson, A. (2004). The hoverflies (Diptera, Syrphidae) of the Madeiran archipelago, Portugal. Dipterists Digest 11, 47–82.

[brv70017-bib-0389] Snow, D. W. & Ross, K. F. A. (1952). Insect migration in the Pyrenees. Entomology Monthly Magazine 88, 1–6.

[brv70017-bib-0390] Southwood, A. & Avens, L. (2010). Physiological, behavioral, and ecological aspects of migration in reptiles. Journal of Comparative Physiology B 180, 1–23. 10.1007/s00360-009-0415-8 19847440

[brv70017-bib-0391] Southwood, T. R. E. & Jepson, W. F. (1962). Studies on the populations of *Oscinella frit* L.(Dipt: Chloropidae) in the oat crop. The Journal of Animal Ecology 31(3), 481–495. 10.2307/2048.

[brv70017-bib-0392] Sparks, A. N. , Jackson, R. D. , Carpenter, J. E. & Muller, R. A. (1986). Insects captured in light traps in the Gulf of Mexico. Annals of the Entomological Society of America 79(1), 132–139. 10.1093/aesa/79.1.132.

[brv70017-bib-0393] Sparks, T. H. , Dennis, R. L. H. , Croxton, P. J. & Cade, M. (2007). Increased migration of Lepidoptera linked to climate change. European Journal of Entomology 104(1), 139–143. 10.14411/eje.2007.019.

[brv70017-bib-0394] Sparks, T. H. , Roy, D. B. & Dennis, R. L. H. (2005). The influence of temperature on migration of Lepidoptera into Britain. Global Change Biology 11(3), 507–514.

[brv70017-bib-0395] * Speight, M. C. D. (1996). A mass migration of *Episyrphus balteatus* and *Eupeodes corollae* arriving in the south‐west and remarks on other migrant hoverflies (Diptera: Syrphidae) in Ireland. Irish Naturalists' Journal 25(5), 182–183.

[brv70017-bib-0396] * Sprygin, A. V. , Fedorova, O. A. , Babin, Y. Y. , Kononov, A. V. & Karaulov, A. K. (2015). Blood‐sucking midges from the genus *Culicoides* (Diptera: Ceratopogonidae) act as filed vectors of human and animal diseases Сельскохозяйственная биология 2 183–197. Sel'skokhozyaistvennaya Biologiya 50(2), 183–197. 10.15389/agrobiology.2015.2.183eng.

[brv70017-bib-0397] * Ssymank, A. , Kearns, C. A. , Pape, T. & Thompson, F. C. (2008). Pollinating flies (Diptera): a major contribution to plant diversity and agricultural production. Biodiversity 9(1–2), 86–89.

[brv70017-bib-0398] Stefanescu, C. , Páramo, F. , Åkesson, S. , Alarcón, M. , Ávila, A. , Brereton, T. , Carnicer, J. , Cassar, L. F. , Fox, R. , Heliölä, J. , Hill, J. K. , Hirneisen, N. , Kjellén, N. , Kühn, E. , Kuussaari, M. , *et al*. (2013). Multi‐generational long‐distance migration of insects: studying the painted lady butterfly in the Western Palaearctic. Ecography 36(4), 474–486.

[brv70017-bib-0399] * Steiner, L. F. , Mitchell, W. C. & Baumhover, A. H. (1962). Progress of fruit‐fly control by irradiation sterilization in Hawaii and the Marianas Islands. International Journal of Applied Radiation and Isotopes 13, 427–433.

[brv70017-bib-0400] * Suh, S. J. & Kwon, Y. J. (2016). First finding of a quarantine pest, *A. therigona* (*A. critochaeta*) *orientalis* Schiner (Diptera: Muscidae), in Korea. Entomological Research 46(3), 185–189.

[brv70017-bib-0401] * Sullivan, M. , Krishna, A. , Krajacich, B. J. & Lehmann, T. (2020). Quantifying flight aptitude variation in wild *Anopheles gambiae* in order to identify long‐distance migrants. Malarial Journal 19(263), 1–15. 10.1186/s12936-020-03333-2 PMC7374819 32698842

[brv70017-bib-0402] * Sullivan, M. S. & Sutherland, J. P. (1999). Geographical variation in morphology and asymmetry in *Episyrphus Balteatus* Degeer (Diptera: Syrphidae). Tijdschrift voor Entomologie 142, 327–331.

[brv70017-bib-0403] * Svensson, B. G. & Janzon, L. Å. (1984). Why does the hoverfly Metasyrphus corollae migrate? Ecological Entomology 9(3), 329–335.

[brv70017-bib-0404] * Tachibana, S. I. & Numata, H. (2006). Seasonal prevalence of blowflies and flesh flies in Osaka City. Entomological Science 9(4), 341–345.

[brv70017-bib-0405] * Tainchum, K. , Duvallet, G. , Akratanakul, P. & Chareonviriyaphap, T. (2009). Genetic diversity and gene flow among stable fly populations, *Stomoxys calcitrans* (L.) in Thailand. Agriculture and Natural Resources 43(3), 526–537.

[brv70017-bib-0406] * Tait, G. , Grassi, A. , Pfab, F. , Crava, C. M. , Dalton, D. T. , Magarey, R. , Ometto, L. , Vezzulli, S. , Rossi‐Stacconi, M. V. , Gottardello, A. & Pugliese, A. (2018). Large‐scale spatial dynamics of *Drosophila suzukii* in Trentino, Italy. Journal of Pest Science 91(4), 1213–1224. 10.1007/s10340-018-0985-x.

[brv70017-bib-0407] Talavera, G. , García‐Berro, A. , Talla, V. N. , Ng'iru, I. , Bahleman, F. , Kébé, K. , Nzala, K. M. , Plasencia, D. , Marafi, M. A. , Kassie, A. & Goudégnon, E. O. (2023). The Afrotropical breeding grounds of the palearctic‐African migratory painted lady butterflies (*Vanessa cardui*). Proceedings of the National Academy of Sciences 120(16), e2218280120. 10.1073/pnas.2218280120 PMC10120051 37036992

[brv70017-bib-0408] Tallamy, D. W. & Shriver, W. G. (2021). Are declines in insects and insectivorous birds related? The Condor 123(1), 1–8.

[brv70017-bib-0409] * Teskey, H. J. (1960). A review of the life‐history and habits of *Musca autumnalis* DeGeer (Diptera: Muscidae). The Canadian Entomologist 92(5), 360–367.

[brv70017-bib-0410] * Thistlewood, H. M. , Rozema, B. & Acheampong, S. (2019). Infestation and timing of use of non‐crop plants by *Drosophila suzukii* (Matsumura)(Diptera: Drosophilidae) in the Okanagan Basin, Canada. The Canadian Entomologist 151(1), 34–48. 10.4039/tce.2018.47.

[brv70017-bib-0411] * Thomas, D. B. & Loera‐Gallardo, J. (1998). Dispersal and longevity of mass‐released, sterilized Mexican fruit flies (Diptera: Tephritidae). Environmental Entomology 27(4), 1045–1052.

[brv70017-bib-0412] * Tirados, I. , Costantini, C. , Gibson, G. & Torr, S. J. (2006). Blood‐feeding behaviour of the malarial mosquito anopheles arabiensis: implications for vector control. Medical and Veterinary Entomology 20(4), 425–437. 17199754 10.1111/j.1365-2915.2006.652.x

[brv70017-bib-0413] * Tomlinson, S. & Menz, M. H. (2015). Does metabolic rate and evaporative water loss reflect differences in migratory strategy in sexually dimorphic hoverflies? Comparative Biochemistry and Physiology Part A: Molecular & Integrative Physiology 190, 61–67. 10.1016/j.cbpa.2015.09.004 26384457

[brv70017-bib-0414] * Townsend, M. T. (1928). Seasonal abundance and vertical migrations of *Lucilia caesar* Linn. And other insects in an artificial grove. Annals of the Entomological Society of America 21(1), 121–131.

[brv70017-bib-0415] Tsacas, L. (1984). Nouvelles données sur la biogéographie et l'évolution du groupe *Drosophila melanogaster* en Afrique. Description de six nouvelles espèces (Diptera, Drosophilidae). Annales de la Société entomologique de France 20, 419–438.

[brv70017-bib-0416] * Tsuda, Y. & Kim, K. S. (2008). Sudden autumnal appearance of adult *Culex tritaeniorhynchus* (Diptera: Culicidae) at a park in urban Tokyo: first field evidence for prediapause migration. Journal of Medical Entomology 45(4), 610–616. 18714859 10.1603/0022-2585(2008)45[610:saaoac]2.0.co;2

[brv70017-bib-0417] * Tsuda, Y. & Kim, K. S. (2010). Prediapause migration and overwintering of *Culex tritaeniorhynchus* (Diptera: Culicidae) observed in a park in urban Tokyo during 2007 to 2009. Medical Entomology and Zoology 61(1), 69–78.

[brv70017-bib-0418] * Tummeleht, L. , Jürison, M. , Kurina, O. , Kirik, H. , Jeremejeva, J. & Viltrop, A. (2020). Diversity of Diptera species in Estonian pig farms. Veterinary Sciences 7(1), 13. 31979423 10.3390/vetsci7010013 PMC7157211

[brv70017-bib-0419] * Ughasi, J. , Bekard, H. E. , Coulibaly, M. , Adabie‐Gomez, D. , Gyapong, J. , Appawu, M. , Wilson, M. D. & Boakye, D. A. (2012). *Mansonia africana* and *Mansonia uniformis* are vectors in the transmission of *Wuchereria bancrofti* lymphatic filariasis in Ghana. Parasites & Vectors 5, 1–5. 22564488 10.1186/1756-3305-5-89 PMC3419659

[brv70017-bib-0420] * Vadivelu, K. (2014). Biology and management of ber fruit fly, *Carpomyia vesuviana* Costa (Diptera: Tephritidae): a review. African Journal of Agricultural Research 9(16), 1310–1317.

[brv70017-bib-0421] * Valido, A. & Olesen, J. M. (2010). Pollination on islands: examples from the Macaronesian archipelagoes. Terrestrial Arthropods of Macaronesia ‐ Biodiversity, Ecology and Evolution, 249–283. Sociedade Portuguesa de Entomologia, Lisbon.

[brv70017-bib-0422] * Van der Goot, V. S. (1964). Summer records of Syrphidae (Diptera) from Sicily, with field notes and descriptions of new species. Zoologische Mededelingen 39(42), 414–432.

[brv70017-bib-0423] * Van der Goot, V. S. (1986). Een opeenhoping van wenkvliegjes op een vreemde plaats (Diptera: Sepsidae). Entomologische Berichten 46(3), 33–34.

[brv70017-bib-0424] * Verdonschot, P. F. & Besse‐Lototskaya, A. A. (2014). Flight distance of mosquitoes (Culicidae): a metadata analysis to support the management of barrier zones around rewetted and newly constructed wetlands. Limnologica 45, 69–79.

[brv70017-bib-0425] * Verheggen, F. , Mignon, J. , Louis, J. , Haubruge, E. & Vanderpas, J. (2008). Mothflies (Diptera: Psychodidae) in hospitals: a guide to their identification and methods for their control. Acta Clinica Belgica 63(4), 251–255. 19048703 10.1179/acb.2008.046

[brv70017-bib-0426] Verhelst, B. , Jansen, J. & Vansteelant, W. (2011). South West Georgia: an important bottleneck for raptor migration during autumn. Ardea 99(2), 137–146.

[brv70017-bib-0427] * Vlasáková, B. , Pinc, J. , Jůna, F. & Kotyková Varadínová, Z. (2019). Pollination efficiency of cockroaches and other floral visitors of *Clusia blattophila* . Plant Biology 21(4), 753–761. 30620429 10.1111/plb.12956

[brv70017-bib-0428] * Vriesekoop, F. & Shaw, R. (2010). The Australian bush fly (*Musca vetustissima*) as a potential vector in the transmission of foodborne pathogens at outdoor eateries. Foodborne Pathogens and Disease 7(3), 275–279. 19895260 10.1089/fpd.2009.0366

[brv70017-bib-0429] * Wang, J. , Li, X. Y. , Du, R. B. & Liu, Y. H. (2021). The complete mitogenome of *Chlorops oryzae* Matsumura (Diptera: Chloropidae). Mitochondrial DNA Part B Resources 6(7), 1844–1846. 34124364 10.1080/23802359.2021.1934171 PMC8183556

[brv70017-bib-0430] * Warmke, H. E. (1952). Studies on natural pollination of *Hevea brasiliensis* in Brazil. Science 116(3018), 474–475. 12994893 10.1126/science.116.3018.474

[brv70017-bib-0431] Warren, T. L. , Giraldo, Y. M. & Dickinson, M. H. (2019). Celestial navigation in *Drosophila* . Journal of Experimental Biology 222(Suppl_1), jeb186148. 30728228 10.1242/jeb.186148 PMC7375828

[brv70017-bib-0432] Weir, P. T. & Dickinson, M. H. (2012). Flying *Drosophila* orient to sky polarization. Current Biology 22(1), 21–27. 22177905 10.1016/j.cub.2011.11.026 PMC4641755

[brv70017-bib-0433] * Weiss, H. B. (1924). Diptera collected on a New Jersey Salt Marsh. The Canadian Entomologist 56(11), 255–257.

[brv70017-bib-0434] Westmacott, H. M. & Williams, C. B. (1954). A migration of Lepidoptera and Diptera in Nepal. Entomologiste 87, 232–234.

[brv70017-bib-0435] * Wharton, R. T. (1962). The biology of *Mansonia* mosquitoes in relation to the transmission of filariasis in Malaya. Bulletins from the Institute for Medical Research, Federated Malay States. 11, 1–114. 14000211

[brv70017-bib-0436] * White, G. B. (1974). *Anopheles gambiae* complex and disease transmission in Africa. Transactions of the Royal Society of Tropical Medicine and Hygiene 68(4), 278–298. 4420769 10.1016/0035-9203(74)90035-2

[brv70017-bib-0437] * Whitfield, F. S. (1939). Air transport, insects and disease. Bulletin of Entomological Research 30(3), 365–442.

[brv70017-bib-0438] Wiegmann, B. M. , Trautwein, M. D. , Winkler, I. S. , Barr, N. B. , Kim, J.‐W. , Lambkin, C. , Bertone, M. A. , Cassel, B. K. , Bayless, K. M. & Heimberg, A. M. (2011). Episodic radiations in the fly tree of life. Proceedings of the National Academy of Sciences 108(14), 5690–5695. 10.1073/pnas.1012675108. PMC3078341 21402926

[brv70017-bib-0439] Wikelski, M. , Moskowitz, D. , Adelman, J. S. , Cochran, J. , Wilcove, D. S. & May, M. L. (2006). Simple rules guide dragonfly migration. Biology Letters 2(3), 325–329. 17148394 10.1098/rsbl.2006.0487 PMC1686212

[brv70017-bib-0440] * Williams, C. B. (1920). Records of insect migration in tropical America. Transactions of the Royal Entomological Society of London 68, 146–165.

[brv70017-bib-0441] Williams, C. B. (1958). Insect Migration, Edition (Volume 36). Collins, London.

[brv70017-bib-0442] Williams, C. B. , Common, I. F. B. , French, R. A. , Muspratt, V. & Williams, M. C. (1956). Observations on the migration of insects in the Pyrenees in the autumn of 1953. Transactions of the Royal Entomological Society of London 108(9), 385–407. 10.1111/j.1365-2311.1956.tb01276.x.

[brv70017-bib-0443] * Williams, F. X. (1944). Biological studies in Hawaiian water‐loving insects, Part 3: Diptera or flies, D. Culicidae, Chironomidae, and Ceratopogonidae. Proceedings of the Hawaiian Entomological Society 12(1), 149–197.

[brv70017-bib-0444] * Williston, S. W. (1884). On the North American Asilidae (Dasypogoninae, Laphrinae), with a new genus of Syrphidae. Transactions of the American Entomological Society and Proceedings of the Entomological Section of the Academy of Natural Sciences 11(1), 1–35.

[brv70017-bib-0445] * Winkler, I. S. , Scheffer, S. J. & Mitter, C. (2009). Molecular phylogeny and systematics of leaf‐mining flies (Diptera: Agromyzidae): delimitation of *Phytomyza* Fallén sensu lato and included species groups, with new insights on morphological and host‐use evolution. Systematic Entomology 34(2), 260–292. 10.1111/j.1365-3113.2008.00462.x.

[brv70017-bib-0446] Wolf, W. W. , Sparks, A. N. , Pair, S. D. , Westbrook, J. K. & Truesdale, F. M. (1986). Radar observations and collections of insects in the Gulf of Mexico. In Insect Flight: Dispersal and Migration, pp. 221–234, Springer, New York.

[brv70017-bib-0447] Wotton, K. R. , Gao, B. , Menz, M. H. M. , Morris, R. K. A. , Ball, S. G. , Lim, K. S. , Reynolds, D. R. , Hu, G. & Chapman, J. W. (2019). Mass seasonal migrations of hoverflies provide extensive pollination and crop protection services. Current Biology 29(13), 2167–2173. 31204159 10.1016/j.cub.2019.05.036

[brv70017-bib-0448] * Wotton, R. S. (1978). Growth, respiration, and assimilation of blackfly larvae (Diptera: Simuliidae) in a lake‐outlet in Finland. Oecologia 33, 279–290. 28309593 10.1007/BF00348114

[brv70017-bib-0449] * Xie, G.‐L. , Ma, X.‐R. , Liu, Q.‐Y. , Meng, F.‐X. , Li, C. , Wang, J. & Guo, Y.‐H. (2021). Genetic structure of *Culex tritaeniorhynchus* (Diptera: Culicidae) based on COI DNA barcodes. Mitochondrial DNA Part B Resources 6(4), 1411–1415. 10.1080/23802359.2021.1911711. 35174283 PMC8843312

[brv70017-bib-0450] * Xu, X. , Coquilleau, M. P. , Ridland, P. M. , Umina, P. A. , Yang, Q. & Hoffmann, A. A. (2021). Molecular identification of leafmining flies from Australia including new *Liriomyza* outbreaks. Journal of Economic Entomology 114(5), 1983–1990. 34279655 10.1093/jee/toab143

[brv70017-bib-0451] * Yaro, A. S. , Linton, Y. M. , Dao, A. , Diallo, M. , Sanogo, Z. L. , Samake, D. , Ousmane, Y. , Kouam, C. , Krajacich, B. J. , Faiman, R. & Bamou, R. (2022). Diversity, composition, altitude, and seasonality of high‐altitude windborne migrating mosquitoes in the Sahel: implications for disease transmission. Frontiers in Epidemiology 2, 1001782. 10.3389/fepid.2022.1001782. 38455321 PMC10910920

[brv70017-bib-0452] * Ye, H. U. I. & Liu, J. H. (2005). Population dynamics of the oriental fruit fly, *Bactrocera dorsalis* (Diptera: Tephritidae) in the Kunming area, southwestern China. Insect Science 12(5), 387–392.

[brv70017-bib-0453] * Yildirim, A. , Dik, B. , Duzlu, O. , Onder, Z. , Ciloglu, A. , Yetismis, G. & Inci, A. (2019). Genetic diversity of Culicoides species within the *Pulicaris* complex (Diptera: Ceratopogonidae) in Turkey inferred from mitochondrial COI gene sequences. Acta Tropica 190, 380–388. 30553894 10.1016/j.actatropica.2018.12.005

[brv70017-bib-0454] Zeng, J. , Liu, Y. , Zhang, H. , Liu, J. , Jiang, Y. , Wyckhuys, K. A. G. & Wu, K. (2020). Global warming modifies long‐distance migration of an agricultural insect pest. Journal of Pest Science 93, 569–581.

[brv70017-bib-0455] * Zuha, R. M. , Ankasha, S. J. , Disney, R. H. L. & Omar, B. (2016). Indoor decomposition study in Malaysia with special reference to the scuttle flies (Diptera: Phoridae). Egyptian Journal of Forensic Sciences 6(3), 216–222.

[brv70017-bib-0456] Караџић, В. С. (2005). Живот и обичаји народа српскога. (Issue 9). Политика.

